# Urine tumor DNA detection of minimal residual disease in muscle-invasive bladder cancer treated with curative-intent radical cystectomy: A cohort study

**DOI:** 10.1371/journal.pmed.1003732

**Published:** 2021-08-31

**Authors:** Pradeep S. Chauhan, Kevin Chen, Ramandeep K. Babbra, Wenjia Feng, Nadja Pejovic, Armaan Nallicheri, Peter K. Harris, Katherine Dienstbach, Andrew Atkocius, Lenon Maguire, Faridi Qaium, Jeffrey J. Szymanski, Brian C. Baumann, Li Ding, Dengfeng Cao, Melissa A. Reimers, Eric H. Kim, Zachary L. Smith, Vivek K. Arora, Aadel A. Chaudhuri

**Affiliations:** 1 Division of Cancer Biology, Department of Radiation Oncology, Washington University School of Medicine, St. Louis, Missouri, United States of America; 2 Division of Medical Oncology, Department of Medicine, Washington University School of Medicine, St. Louis, Missouri, United States of America; 3 Siteman Cancer Center, Barnes Jewish Hospital and Washington University School of Medicine, St. Louis, Missouri, United States of America; 4 Department of Genetics, Washington University School of Medicine, St. Louis, Missouri, United States of America; 5 McDonnell Genome Institute, Washington University School of Medicine, St. Louis, Missouri, United States of America; 6 Division of Biology and Biomedical Sciences, Washington University School of Medicine, St. Louis, Missouri, United States of America; 7 Department of Pathology and Immunology, Washington University School of Medicine, St. Louis, Missouri, United States of America; 8 Division of Urology, Department of Surgery, Washington University School of Medicine, St. Louis, Missouri, United States of America; 9 Department of Biomedical Engineering, Washington University School of Medicine, St. Louis, Missouri, United States of America; 10 Department of Computer Science and Engineering, Washington University in St. Louis, St. Louis, Missouri, United States of America

## Abstract

**Background:**

The standard of care treatment for muscle-invasive bladder cancer (MIBC) is radical cystectomy, which is typically preceded by neoadjuvant chemotherapy. However, the inability to assess minimal residual disease (MRD) noninvasively limits our ability to offer bladder-sparing treatment. Here, we sought to develop a liquid biopsy solution via urine tumor DNA (utDNA) analysis.

**Methods and findings:**

We applied urine Cancer Personalized Profiling by Deep Sequencing (uCAPP-Seq), a targeted next-generation sequencing (NGS) method for detecting utDNA, to urine cell-free DNA (cfDNA) samples acquired between April 2019 and November 2020 on the day of curative-intent radical cystectomy from 42 patients with localized bladder cancer. The average age of patients was 69 years (range: 50 to 86), of whom 76% (32/42) were male, 64% (27/42) were smokers, and 76% (32/42) had a confirmed diagnosis of MIBC. Among MIBC patients, 59% (19/32) received neoadjuvant chemotherapy. utDNA variant calling was performed noninvasively without prior sequencing of tumor tissue. The overall utDNA level for each patient was represented by the non-silent mutation with the highest variant allele fraction after removing germline variants. Urine was similarly analyzed from 15 healthy adults. utDNA analysis revealed a median utDNA level of 0% in healthy adults and 2.4% in bladder cancer patients. When patients were classified as those who had residual disease detected in their surgical sample (*n* = 16) compared to those who achieved a pathologic complete response (pCR; *n* = 26), median utDNA levels were 4.3% vs. 0%, respectively (*p* = 0.002). Using an optimal utDNA threshold to define MRD detection, positive utDNA MRD detection was highly correlated with the absence of pCR (*p* < 0.001) with a sensitivity of 81% and specificity of 81%. Leave-one-out cross-validation applied to the prediction of pathologic response based on utDNA MRD detection in our cohort yielded a highly significant accuracy of 81% (*p* = 0.007). Moreover, utDNA MRD–positive patients exhibited significantly worse progression-free survival (PFS; HR = 7.4; 95% CI: 1.4–38.9; *p* = 0.02) compared to utDNA MRD–negative patients. Concordance between urine- and tumor-derived mutations, determined in 5 MIBC patients, was 85%. Tumor mutational burden (TMB) in utDNA MRD–positive patients was inferred from the number of non-silent mutations detected in urine cfDNA by applying a linear relationship derived from The Cancer Genome Atlas (TCGA) whole exome sequencing of 409 MIBC tumors. We suggest that about 58% of these patients with high inferred TMB might have been candidates for treatment with early immune checkpoint blockade. Study limitations included an analysis restricted only to single-nucleotide variants (SNVs), survival differences diminished by surgery, and a low number of DNA damage response (DRR) mutations detected after neoadjuvant chemotherapy at the MRD time point.

**Conclusions:**

utDNA MRD detection prior to curative-intent radical cystectomy for bladder cancer correlated significantly with pathologic response, which may help select patients for bladder-sparing treatment. utDNA MRD detection also correlated significantly with PFS. Furthermore, utDNA can be used to noninvasively infer TMB, which could facilitate personalized immunotherapy for bladder cancer in the future.

## Abstract 2

Pradeep S. Chauhan and colleagues, investigate a liquid biopsy solution via urine tumor DNA (utDNA) analysis to assess minimal residual disease in patients with muscle-invasive bladder cancer.

## Author summary

**Why was this study done?:**

- The standard of care for muscle-invasive bladder cancer (MIBC) is neoadjuvant chemotherapy followed by radical cystectomy, which significantly impacts quality of life.
- The inability to assess minimal residual disease (MRD) noninvasively limits our ability to offer bladder-sparing treatment.
- We determine if urine tumor DNA (utDNA) detection just prior to radical cystectomy can predict pathologic complete response (pCR) and differences in survival outcomes.
- We determine if utDNA analysis can identify patients who are candidates for early immune checkpoint blockade.

**What did the researchers do and find?:**

- We applied urine Cancer Personalized Profiling by Deep Sequencing (uCAPP-Seq) to urine cell-free DNA (cfDNA) samples acquired on the day of curative-intent radical cystectomy from 42 patients with localized bladder cancer.
- Positive utDNA MRD detection was highly correlated with the absence of pCR (*p* < 0.001) with a sensitivity of 81% and specificity of 81%.
- utDNA MRD–positive patients exhibited significantly worse progression-free survival (PFS) compared to utDNA MRD–negative patients (HR = 7.4; 95% CI: 1.4–38.9; *p* = 0.02).
- Patients with high TMB inferred from urine might have been candidates for early treatment with immune checkpoint blockade.

**What do these findings mean?:**

- utDNA MRD detection prior to curative-intent radical cystectomy for bladder cancer correlated significantly with pathologic response, which may help select patients for bladder-sparing treatment.
- utDNA can be used to noninvasively infer TMB, which could facilitate personalized immunotherapy for bladder cancer patients in the future.

## Introduction

In the United States of America, bladder cancer is the sixth most commonly diagnosed cancer overall and the fourth most commonly diagnosed malignancy in men [1]. Clinical management is largely dependent on the extent to which the tumor penetrates the bladder wall at time of presentation [2]. Approximately 70% of patients initially present with non–muscle-invasive bladder cancer (NMIBC) [3]. These patients are typically treated with transurethral resection of bladder tumor alone or in conjunction with intravesical therapy. While 5-year survival rates for NMIBC are favorable, patients carry a high risk of recurrence or progression to more advanced stages of disease [4]. Therefore, these patients require frequent monitoring with invasive cystoscopic examinations. As a result, bladder cancer has been found to have the highest lifetime cost per patient of any malignancy [5,6].

Approximately 25% of bladder cancer patients present with organ-confined muscle-invasive bladder cancer (MIBC), characterized by tumor that extends to the underlying detrusor muscle [7]. The standard of care treatment recommendation for both MIBC and recurrent/progressive NMIBC after appropriate intravesical therapy is radical cystectomy with urinary diversion, a significant surgical procedure with a major impact on quality of life [8,9]. For MIBC patients, this is often preceded by cisplatin-based neoadjuvant chemotherapy [10]. Despite these aggressive measures, the overall 5-year survival for MIBC is still only about 50%, and the recurrence risk is substantial [11].

Pathologic response is a strong prognostic indicator of long-term survival after radical cystectomy [12,13]. Based on evaluation of the radical cystectomy specimen, approximately 35% of patients will achieve a pathologic complete response (pCR) following neoadjuvant treatment [10,14]. Retrospective data support the idea that some patients who achieve an excellent response to neoadjuvant treatment may be able to safely forgo radical cystectomy [15]. If patients likely to have achieved a pCR could be better identified prospectively, this could support personalized care, enabling some patients to avoid the life-altering morbidity of urinary diversion and potential mortality from operative complications [8].

Cystoscopy, while considered invasive and uncomfortable, can be used to evaluate tumor response to neoadjuvant chemotherapy or chemoradiation. However, cystoscopy prior to radical cystectomy frequently under-stages disease burden [16,17], creating a major barrier to testing and implementing personalized bladder-sparing treatment paradigms. Furthermore, patients managed with bladder-sparing treatment remain at significant risk for recurrence [15], thus requiring frequent monitoring with invasive cystoscopy [17]. Therefore, it is critical that we advance our standard of care to enable improved accuracy and reduced morbidity when assessing and monitoring localized bladder cancer.

Plasma- and urine-based biomarkers present a promising opportunity for noninvasive management of localized bladder cancer [18,19]. Cancer Personalized Profiling by Deep Sequencing (CAPP-Seq) with integrated digital error suppression (iDES) is an ultrasensitive method of high-throughput sequencing used to detect circulating tumor DNA (ctDNA) [20,21]. In recent years, the utility of CAPP-Seq with iDES in the detection of minimal residual disease (MRD) has been validated in a variety of tumor types [22–28]. Urine tumor DNA (utDNA) analysis with an adapted version of CAPP-Seq, called urine Cancer Personalized Profiling by Deep Sequencing (uCAPP-Seq), was recently shown to be a highly sensitive urine-based approach for monitoring NMIBC patients treated conservatively with modalities other than radical cystectomy [28].

Here, we seek to validate uCAPP-Seq by applying it to urine collected from a largely MIBC cohort with every patient treated with curative-intent radical cystectomy. We determine if utDNA variant calling without prior sequencing of tumor tissue using urine samples collected just prior to radical cystectomy can predict pathologic staging and differences in survival outcomes, namely progression-free survival (PFS) and overall survival (OS). Furthermore, we address the hypothesis that utDNA can be used to infer tumor mutational burden (TMB) and identify actionable mutations, which could potentially inform personalized adjuvant treatment including immune checkpoint blockade and targeted systemic therapy for utDNA MRD–positive patients.

## Methods

### Study design

**Fig 1:**
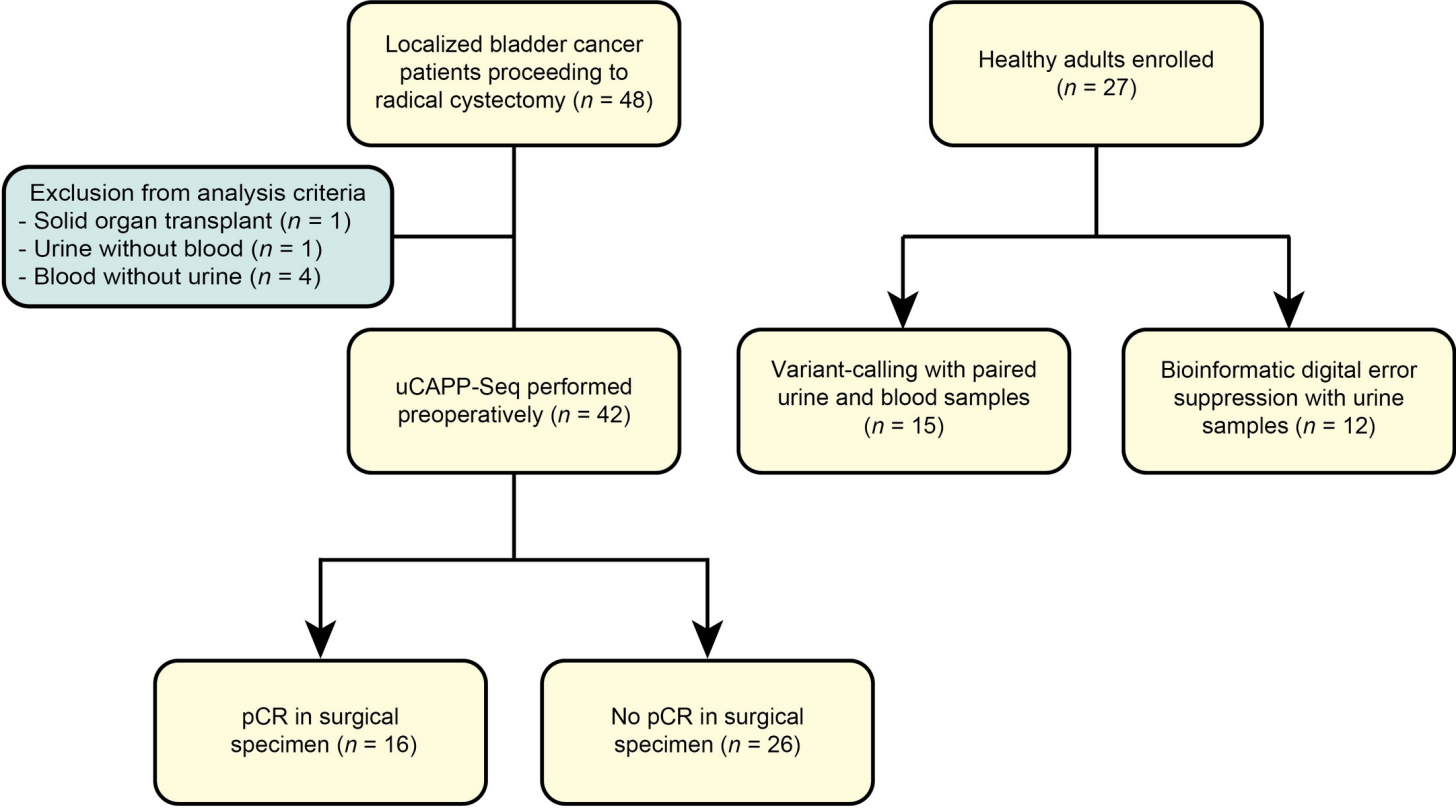
Patient enrollment and sample collection for utDNA MRD analysis. Patients with localized bladder cancer who were candidates for radical cystectomy were enrolled onto this study, as were healthy adult donors. Biofluid samples were then collected for utDNA analysis as shown in the schema. MRD, minimal residual disease; pCR, pathologic complete response; uCAPP-Seq, urine Cancer Personalized Profiling by Deep Sequencing; utDNA, urine tumor DNA.

Our cohort study was designed to determine whether utDNA on the day of radical cystectomy correlates with pathologic response and detects MRD in patients with localized bladder cancer. Thus, we enrolled 48 patients with localized bladder cancer undergoing curative-intent radical cystectomy and 27 healthy adults at Washington University from April 2019 to November 2020 (**Fig 1**). Patients and healthy adults were enrolled onto institutionalized human research protocols approved by the Human Research Protection Office at Washington University (Institutional Review Board [IRB] #201411135 and IRB #201903142, respectively) and provided written informed consent to the collection and molecular analysis of biological specimens (urine, blood, and/or tumor tissue) in accordance with the Declaration of Helsinki. The protocol can be accessed on ClinicalTrials.gov (NCT04354064). We profiled and analyzed paired urine and blood samples acquired on the day of radical cystectomy from 42 consented patients (**S1 Table**). Patients were excluded if they had a blood sample without urine, a urine sample without blood, or a history of immunosuppression for a solid organ transplant, which has been shown to confound the detection of tumor DNA [29,30]. In 2 patients, we additionally obtained serial urine samples prior to and during neoadjuvant chemotherapy. Patients continued clinical follow-up after radical cystectomy as per the standard of care, and the managing clinicians were blinded to the utDNA data. Paired urine and blood samples were also collected from 15 healthy adults for variant calling analysis. Urine samples without blood were collected from another 12 healthy adults and used to reduce stereotypical base substitution errors for our serial mutation tracking and TMB analyses (see Methods: utDNA monitoring during neoadjuvant chemotherapy and Methods: Inferring TMB from utDNA analysis) [21]. This study is reported as per the Strengthening the Reporting of Observational Studies in Epidemiology (STROBE) guideline (S1 Checklist).

### Eligibility criteria

Patients were required to have a histologic or cytologic diagnosis of bladder cancer amenable for radical cystectomy. MIBC was defined as invasive urothelial carcinoma staged at least T2 or higher (i.e., invasion of the detrusor muscle) found on transurethral resection of bladder tumor or radiographically determined by a board-certified urologic oncologist and/or medical oncologist. In some equivocal cases, a multidisciplinary tumor board assessment was required to determine a case for surgical resection. The primary bladder cancer could have been previously treated with intravesical agents such as Bacillus Calmette–Guérin (BCG), valrubicin, or gemcitabine. Some cases of T1 disease were also allowed into the study. The decision to proceed with cystectomy in cases of T1 disease was made by the patient and the treating physician, often in collaboration with a multidisciplinary tumor board. These cases were resistant to prior intravesical chemotherapy and/or considered at high risk for progression based on pathological features. We sought to include any bladder cancer patient with an indication for radical cystectomy into our study in order to extend the application of predicting pathologic response based on utDNA to a broader, real-world patient population that could potentially benefit from bladder-sparing treatment. Patients with any other known active cancer diagnoses other than bladder cancer were excluded from enrollment. Electronic medical records were reviewed for each patient to ensure that they met the eligibility criteria for this study.

### Pathologic response assessment

All surgical samples were sampled consistently following standard collection, handling, and submission procedures. The resected surgical specimens (e.g., bladder, prostate, and pelvic lymph nodes) were submitted to the Pathology Department at Washington University for review by a genitourinary surgical pathologist. Based on the eligibility criteria used in an enrolling multi-institutional Alliance trial on nonmetastatic MIBC (NCT03609216) [31], we defined pCR as stages T0, Tis, and/or Ta with no involved lymph nodes in the surgical specimen. This is consistent with the definition used in other solid tumor malignancies as well, including breast cancer [32]. An absence of pCR was defined as stages T1 to T4 or evidence of nodal/metastatic disease in the surgical specimen based on the American Joint Committee on Cancer (AJCC) 8th edition criteria [33].

### Urine cell-free DNA stability in the presence of EDTA

Multiple urine samples from a healthy adult were used to test the stability of urine cell-free DNA (cfDNA) in the presence of EDTA (**S1 Fig**). Pooled urine from this individual was aliquoted into 4 pairs of two 10-mL replicates, each pair testing urine cfDNA stability at different time points (0, 1, 3, and 7 days), with or without EDTA. Thus, 10 mM EDTA was added to one aliquot, and an equal amount of nuclease-free water was added to the other aliquot. The urine samples were then stored at room temperature (approximately 20°C) for these different lengths of times. After each time point, the urine was centrifuged, and the supernatant was transferred to fresh tubes and stored at −80°C. Urine cfDNA was isolated from the stored supernatant. Urine cfDNA was purified further by AMPure XP (Beckman Coulter Life Sciences, Indianapolis, Indiana, USA) magnetic bead–based size selection. We removed genomic DNA using 0.6× concentration of beads, followed by 1.8× concentration of beads to capture the remaining urine cfDNA. Isolated urine cfDNA was analyzed by electropherogram obtained using an Agilent 2100 Bioanalyzer (Agilent Technologies, Santa Clara, California, USA).

### Comparison of urine cell-free DNA fragmentation with in silico size selection

We compared 2 different methodologies for optimizing the detection of variants prior to urine cfDNA library preparation. Urine cfDNA was isolated with Q-sepharose from 4 MIBC patients, as described in the “Urine cell-free DNA isolation and quantification” section below, and processed using 1 of 2 methodologies: standard whole urine cfDNA fragmentation or in vitro size selection (**S2 Fig**). For the fragmentation method, we acoustically sheared urine cfDNA to approximately 200-bp fragments using a LE220 focused ultrasonicator (Covaris, Woburn, Massachusetts, USA). For the in vitro size selection protocol, we used AMPure XP magnetic beads to enrich cfDNA fragments ranging from 70 to 450 bp before sequencing. Following each method, we prepared sequencing libraries per the uCAPP-Seq protocol. We compared the median deduplicated depth (**S3A Fig**), number of non-silent mutations detected (**S3B Fig**), and variant allele fraction levels (**S3C Fig**) among the common non-silent mutations found by both methodologies. Only variants with duplex support were considered in this analysis.

### Biological specimen collection and processing

Urine samples, ranging in volume between 22 and 90 mL, from 42 localized bladder cancer patients were collected in sterile vials containing 2 mL of 0.5 M EDTA in the preoperative setting. Samples were centrifuged at 2,000 g for 10 minutes at room temperature (approximately 20°C). After centrifugation, supernatant was transferred to a fresh tube and then stored at −80°C. Along with urine, patient blood was collected in K2-EDTA Vacutainer tubes (Becton Dickinson, Franklin Lakes, New Jersey, USA), from which germline plasma-depleted whole blood was collected and stored at −80°C as previously described [23,34]. Tumor tissue was also available for sequencing from 5 patients with no antecedent systemic therapy. Tumor tissue was acquired either from the radical cystectomy or transurethral resection of bladder tumor and was either stored fresh-frozen or processed as formalin-fixed paraffin-embedded samples.

### Urine cell-free DNA isolation and quantification

Urine cfDNA was prepared from 22 to 90 mL of urine with Q-sepharose resin slurry (GE Healthcare, Chicago, Illinois, USA) at a ratio of 10 μL slurry per mL of urine and mixed as previously described [28,35]. After 30 minutes, the urine/resin mixture was centrifuged for 10 minutes at 1,800 g. The supernatant was discarded, and resin was washed twice with 0.3 M LiCl/10 mM sodium acetate (pH 5.5), applying 2 mL per 100-μL resin. The resin was then transferred to a Micro Bio-Spin column (Bio-Rad, Hercules, California, USA), and the bound material was eluted by adding 3 separate 670-μL aliquots of 2 M LiCl/10 mM sodium acetate (pH 5.5). Next, the eluates were combined in 70% ethanol and passed over a QIAquick column (Qiagen, Hilden, Germany). The column was washed with 5 mL of 2 M LiCl in 70% ethanol, followed by 5 mL of 75 mM potassium acetate (pH 5.5) in 80% ethanol. We removed residual liquid by centrifuging the columns at 20,000 g for 3 minutes. Finally, bound DNA was eluted into 50 μL of nuclease-free water or 10 mM Tris-Cl (pH 8.5). DNA concentrations were measured using a Qubit dsDNA HS Assay Kit and Qubit 4.0 Fluorometer (Thermo Fisher Scientific, Waltham, Massachusetts, USA).

### Germline and tumor DNA isolation and quantification

The QIAamp DNA Micro Kit (Qiagen) was employed to extract germline DNA from 100 μL of plasma-depleted whole blood or tumor DNA from freshly frozen tumor tissue. The AllPrep DNA/RNA FFPE Kit (Qiagen) was used to extract DNA from one tumor-embedded paraffin block according to the manufacturer’s instructions. DNA was then quantified using the Qubit assay per the manufacturer’s recommendations (Thermo Fisher Scientific).

### DNA library construction and sequencing

Urine cfDNA was fragmented to approximately 200-bp size, while germline DNA and tumor DNA were sheared to approximately 250-bp size prior to library preparation using a LE220 Focused Ultrasonicator (Covaris). All urine cfDNA and germline DNA libraries were prepared using the Kapa HyperPrep Kit with custom sequencing adapters containing demultiplexing, deduplicating, and duplex barcodes [21]. Minimum inputs of 32 ng were used for each library preparation reaction. Targeted hybrid-capture was performed per the uCAPP-Seq protocol [28] with the following modifications: For urine cfDNA, 3 or 4 samples were captured with the custom bladder cancer selector panel (**S2 and S3 Tables**), while 6-plex captures and 12-plex captures were performed for tumor and germline samples, respectively. Libraries were sequenced on an Illumina HiSeq 4000 with 2 × 150 bp paired-end reads. Sequencing quality control metrics including deduplicated depth, on-target rate, fragment size, and duplex recovery rate were reviewed (**S4–S6 Tables**). We sequenced urine cfDNA from localized bladder cancer patients and healthy donors to a median deduplicated depth of 811×, while germline DNA and tumor DNA were sequenced to a median deduplicated depth of 781× and 1917×, respectively (**S4–S6 Tables**). Sequences were analyzed for single-nucleotide variants (SNVs) using the uCAPP-seq bioinformatics pipeline with error suppression [20,21]. Briefly, sequencing reads were demultiplexed using sample-level index barcodes, mapped to the reference genome GRCh37/hg19 (February 2009), filtered for properly paired reads, filtered for bases with Phred quality score ≥30, and deduplicated using unique molecular barcodes. The molecular barcoding strategy enabled the identification of duplex-supported reads (**S4 and S5 Tables**).

### Duplex-supported variant calling for utDNA MRD analysis

utDNA was assessed by deep targeted sequencing of preoperative urine-derived cfDNA. Notably, utDNA analysis from urine was performed without prior sequencing of tumor tissue (i.e., tumor-naive approach). We used a focused MRD gene panel encompassing 49 consensus driver genes frequently mutated in bladder cancer [36–40] (**S2 Table**). For SNV calling from urine, we utilized the CAPP-Seq with iDES pipeline [20,21,28] and filtered out any mutation also detected in the matched germline DNA sample. SNVs with population frequency >0.0001 in the Genome Aggregation Database (gnomAD) were also filtered out [41]. The identified variants were further annotated with ANNOVAR [42] for mutation type, associated gene name, location with respect to associated gene, and amino acid change when relevant, and only those with duplex support were considered further for the MRD analysis. These duplex-supported SNVs were then categorized as silent versus non-silent mutations. Silent mutations included the following: exonic synonymous mutations, intronic mutations (except in *PLEKHS1*), promoter mutations (except in *TERT*), 5′ UTR mutations (except in *TBC1D12*), and 3′ UTR mutations. Non-silent mutations included the following: exonic nonsynonymous mutations, splicing mutations, stop codon mutations, and the aforementioned exceptions based on evidence in the literature [28,36,38]. Overall utDNA levels were finally quantified as the highest variant allele fraction among non-silent mutations with duplex support detected by CAPP-Seq per urine sample. We required duplex-supported reads in our MRD variant calling approach in order to significantly reduce the background error rate as previously described [21].

We bioinformatically tested our MRD gene panel, spanning 145 kb and consisting of 49 consensus driver genes, on 2 whole exome sequencing datasets of pretreatment MIBC tumors: from The Cancer Genome Atlas (TCGA, *n* = 409) and from both the Dana-Farber Cancer Institute and Memorial Sloan Kettering Cancer Center (DFCI/MSKCC, *n* = 50) [38,39]. Concordance between urine cfDNA variants and tumor tissue variants was also assessed in 5 patients with paired urine and tumor tissue available. Combined lists of non-silent mutations with duplex support identified in urine or tumor were queried among non-reference variants present in tumor and urine, respectively (**S7 Table**).

### utDNA monitoring during neoadjuvant chemotherapy

We also obtained serial urine samples from 2 patients prior to starting neoadjuvant chemotherapy, while on neoadjuvant chemotherapy, and on the day of surgery. While duplex-supported reads were not required for variant monitoring, we utilized background polishing using 12 healthy donor urine samples to reduce stereotypical base substitution errors as previously described [21,28]. We combined all the mutations detected after iDES and then monitored each mutation at every time point using the CAPP-Seq bioinformatic pipeline as previously described [20,21,28].

### Inferring TMB from utDNA analysis

TMB was defined as the total number of non-silent mutations in the whole exome. We utilized an expanded custom hybrid-capture panel (called “TMB panel” in this study) to infer TMB in our cohort. The TMB panel is 387 kb in size, covers 536 genes, and spans all regions in the previously published uCAPP-Seq panel [28] along with canonical DNA damage response (DDR) genes [43] (**S3 Table**). To establish the relationship between the number of mutations observed in our uCAPP-Seq TMB panel to exome-wide TMB in bladder cancer, we downloaded somatic variant calls from 409 MIBC tumors that underwent whole exome sequencing by TCGA from https://www.cbioportal.org/ [44]. Non-silent mutations in TCGA whole exome sequencing data were defined as the following mutation types: frameshift mutation (deletion/insertion), missense mutation, nonsense mutation, nonstop mutation, and splice mutation (region/site). We filtered out in-frame mutations (deletion/insertion) and mutations in the 3′ flank, 3′ UTR, 5′ flank, 5′ UTR, and intronic regions. TMB panel versus whole exome sequencing mutational loads were compared using Pearson correlation. We used this simple linear regression equation to infer the TMB levels from urine for all utDNA MRD–positive patients in our cohort, which we applied after accounting for potential dropout in utDNA results as previously described [20,21].

### Identifying actionable mutations from utDNA analysis

utDNA variants detected within our MRD gene panel after uCAPP-Seq were queried in the OncoKB database [45] to identify patients who may harbor a clinically actionable mutation (**S8 Table**). Level 3A evidence denotes compelling clinical data supporting this mutation as a biomarker predictive of response to a drug in bladder cancer [45]. Level 3B evidence denotes a mutation as a biomarker predictive of response to a Food and Drug Administration (FDA)-approved drug or an investigational drug in another malignancy [45].

### Power and statistical analyses

Previous clinical studies in MIBC have shown that patients who do not attain a pCR experience significantly increased risk of progression and death due to residual disease compared to patients with pCR [12,13]. Thus, we hypothesized a large difference in utDNA levels between patients with pCR compared to those without pCR. Based on a large effect size estimated by Cohen’s *f* = 0.5, we require at least 14 subjects in each group to detect a difference with 80% power, as determined by a 1-way ANOVA with a significance level of 0.05. Therefore, we collected paired urine and blood samples from healthy donors and bladder cancer patients with enrollment until we reached at least 14 subjects for each subgroup of the analysis.

Patient characteristics were statistically compared between groups of pCR and no pCR patients using Fisher exact test for categorical variables, Student *t* test for normally distributed continuous variables, and Mann–Whitney U test for non-normally distributed continuous variables. Normality was assessed using the Shapiro–Wilk test with a 0.05 significance level.

utDNA levels in each group of cohort subjects represented distributions of continuous variables and were compared without assuming normality using the Mann–Whitney U test with a 0.017 significance level, adjusted with a Bonferroni correction for comparison of 3 groups. For our analysis restricted to patients who received neoadjuvant chemotherapy, utDNA levels were compared using the Mann–Whitney U test with a 0.05 significance level. To assess utDNA as a classifier of pathologic response, we performed receiver operating characteristic (ROC) analysis and measured the area under the curve. MRD detection was determined by identifying the utDNA level threshold on the ROC curve that classified patients with no pCR versus healthy adults with the highest Youden index. Then, we applied this optimal threshold (utDNA level of 2.3%) to establish utDNA MRD detection among patients with pCR or no pCR. Fisher exact test with a 0.017 significance level, adjusted with a Bonferroni correction for comparison of multiple proportions, was used to assess the association between utDNA MRD detection and pathologic response.

Leave-one-out cross-validation was performed in *R* using the *caret* package’s *LOOCV* method to assess the generalizability of our utDNA MRD predictor to independent data. This was done by generating a univariate logistic regression to predict pCR based on utDNA MRD from k-1 samples and applying this model to predict the kth sample over k iterations. Accuracy of prediction was determined using the *confusionMatrix* function with *p*-value computed using the 1-sided binomial test function.

Kaplan–Meier analysis with log-rank test was performed for PFS and OS based on follow-up within 200 days of surgery. Multivariate logistic regression analysis was performed using the *glm* function in *R* for predicting the absence of pCR using the following covariates: utDNA MRD detection, smoking status, neoadjuvant chemotherapy, staging at the time of diagnosis or transurethral resection of bladder tumor, age (continuous variable), sex, ethnicity, and histology. All statistical analyses were performed using *R* v3.6.3 (http://www.r-project.org) through the RStudio v1.1.463 environment (RStudio, Boston, Massachusetts, USA) and Prism 8 (GraphPad Software, San Diego, California, USA).

## Results

### Cohort characteristics

**Fig 2:**
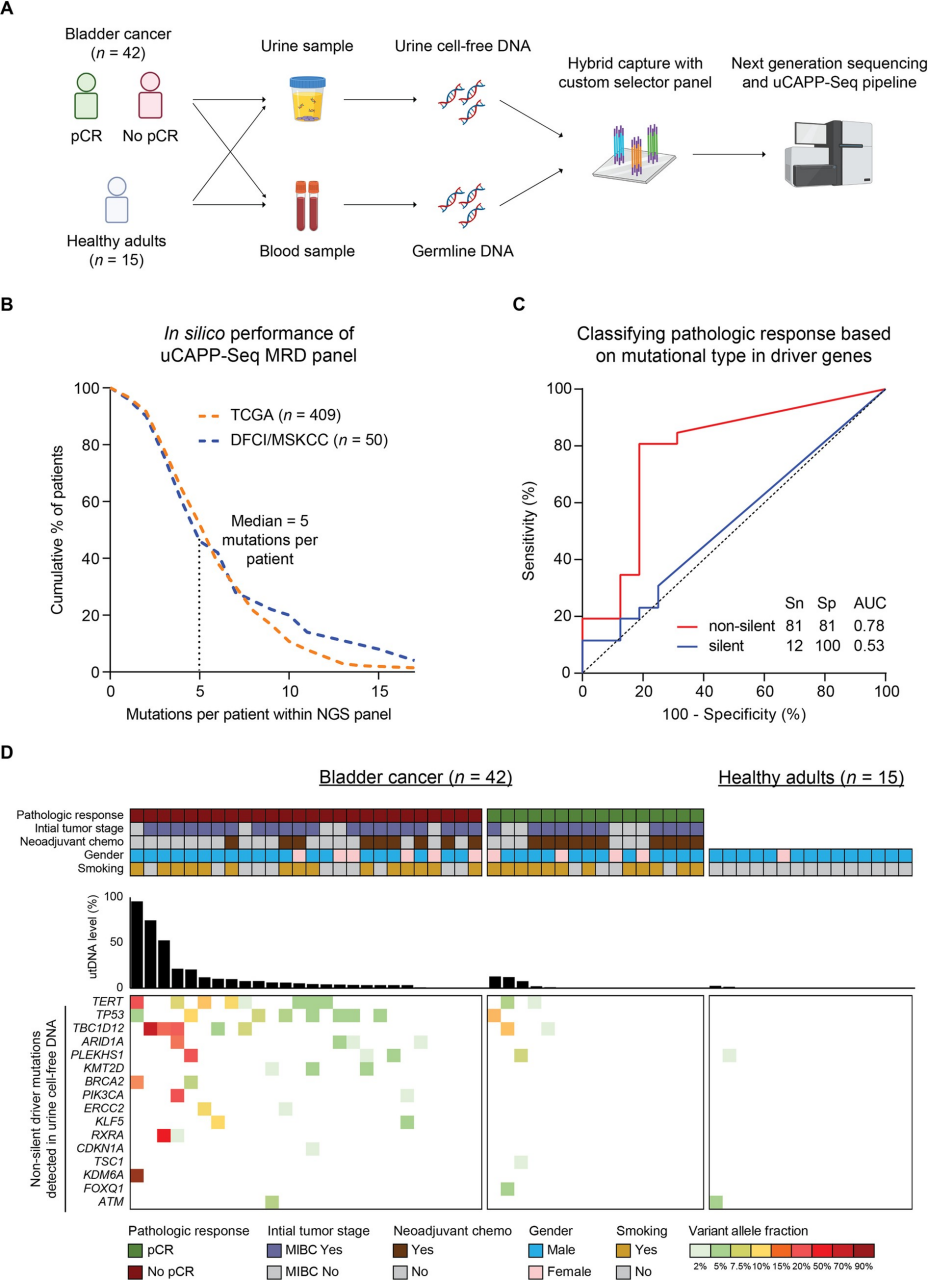
uCAPP-Seq application of MRD gene panel to urine samples. **(A)** Study schema. Urine and blood samples from 42 localized bladder cancer patients scheduled for radical cystectomy and 15 healthy adults were profiled by uCAPP-Seq. Samples from localized bladder cancer patients were collected preoperatively on the day of surgery. Sequencing libraries were prepared from urine cfDNA and peripheral blood germline DNA. utDNA analysis and results were primarily correlated with pathologic response in this study. **(B)** In silico application of the MRD panel, consisting of 49 consensus driver genes, to MIBC cases characterized by TCGA (*n* = 409) and DFCI/MSKCC (*n* = 50). A median of 5 mutations (silent and non-silent) per patient was detected in both datasets as indicated. **(C)** ROC curve classified pCR (*n* = 16) from no pCR (*n* = 26) patients by utDNA level, represented by the mutation with highest VAF. utDNA level derived from non-silent mutations (red curve) classified pathologic response significantly more accurately than silent mutations (blue curve). **(D)** Co-mutation plot showing non-silent driver mutations (with duplex support) detected in the urine of each patient with no pCR, patient with pCR, and healthy adult (bottom heatmap). utDNA level per variant is depicted within the heatmap, while the conglomerate level per subject is represented by the bar graph. Key patient characteristics are represented by the top heatmap. AUC, area under the curve; cfDNA, cell-free DNA; DFCI/MSKCC, Dana-Farber Cancer Institute and Memorial Sloan Kettering Cancer Center; MIBC, muscle-invasive bladder cancer; MRD, minimal residual disease; NGS, next-generation sequencing; pCR, pathologic complete response; ROC, receiver operating characteristic; Sn, sensitivity; Sp, specificity; TCGA, The Cancer Genome Atlas; uCAPP-Seq, urine Cancer Personalized Profiling by Deep Sequencing; utDNA, urine tumor DNA; VAF, variant allele fraction.

We profiled urine cfDNA and matched peripheral blood germline samples collected from 42 patients with localized bladder cancer and 15 healthy donors (**Fig 2A**). Our bladder cancer cohort was comprised of 76% (32/42) males and 64% (27/42) smokers, and the average age was 69 years. A total of 76% (32/42) of patients had a confirmed diagnosis of MIBC, 59% of whom received neoadjuvant chemotherapy, while the remaining 24% had high-risk NMIBC (**Table 1, S1 Table**). No patients had any other known active cancer diagnoses at the time of surgery.

**Table 1:** Characteristics of enrolled bladder cancer patients and healthy adults.

Characteristics	Number	Percentage
**Bladder cancer patients (*n* = 42)**		
**Sex**, *n* (%)		
Male	32	76
Female	10	24
**Age** (years)	69 (50–86)	_
**Ethnicity**, *n* (%)		
White	40	95
Non-White	2	5
**Follow-up time** (days)	183 (27–200)	_
**Smoking**, *n* (%)		
Yes	27	64
No	15	35
Pack-years	20 (0–56)	_
**Pretreatment T stage**^a^, *n* (%)		
Ta	2	5
Tis	1	2
T1	7	17
T2	26	62
T3	6	14
T4	0	0
**Neoadjuvant chemotherapy received**, *n* (%)		
Yes	19	45
ddMVAC	4	21
Gemcitabine/cisplatin	10	53
Gemcitabine/carboplatin	1	5
Carboplatin/paclitaxel	2	11
Treatment switch^b^	1	5
Unknown regimen type	2	11
No	23	55
**pCR**^c^, *n* (%)	16	37
**Histology**, *n* (%)		
Urothelial	36	85
Squamous	3	7
Other	3	7
**Healthy adults** ^**d**^ **(*n* = 27)**		
**Sex**, *n* (%)		
Male	22	81
Female	5	19
**Age** (years)	31 (20–65)	_

^a^T staging was performed at the time of pretreatment TURBT using the AJCC 8 criteria.

^b^Treatment switch: 1 cycle of carboplatin/paclitaxel and 3 cycles of gemcitabine/carboplatin (switched due to cutaneous reaction to paclitaxel).

^c^pCR was defined as T0N0, TaN0, or TisN0 (see Methods: Pathologic response assessment).

^d^Urine and peripheral blood samples from 15 healthy adults were collected to assess specificity of the variant calling approach (see Figs 1 and 2A and Methods: Study design). Urine samples were collected from another 12 healthy adults to use for iDES (see Fig 1, Methods: Study design, and Methods: utDNA monitoring during neoadjuvant chemotherapy).

AJCC 8, American Joint Committee on Cancer 8th edition; ddMVAC, dose-dense methotrexate, vinblastine, doxorubicin, and cisplatin; iDES, integrated digital error suppression; pCR, pathologic complete response; TURBT, transurethral resection of bladder tumor.

### Optimization of urine cell-free DNA processing

All patient urine samples were collected preoperatively just prior to the time of radical cystectomy with the addition of EDTA to preserve the stability of urine cfDNA at room temperature (**S1 Fig**). We also compared 2 different methodologies of urine cfDNA processing prior to urine cfDNA library preparation: standard protocol of acoustic fragmentation versus in vitro size selection of smaller fragments (**S2 Fig**). Although we achieved significantly higher median deduplicated depth (**S3A Fig**) with the size selection method compared to the fragmentation protocol in 4 MIBC patients, there were no significant differences in the number of non-silent mutations detected (**S3B Fig**) or in the variant allele fraction levels among common non-silent mutations (**S3C Fig**) detected by the 2 techniques.

### Development and validation of MRD gene panel for utDNA detection

We used a noninvasive approach without prior sequencing of tumor tissue using uCAPP-Seq to profile mutations detected in urine. For a targeted evaluation of defined consensus regions, we used a focused MRD gene panel encompassing 49 consensus driver genes frequently mutated in bladder cancer (**S2 Table**). These driver genes were selected from previous literature with comprehensive genomic characterization of bladder cancer [36–40]. In silico application of this MRD gene panel to 409 MIBC tumors profiled by TCGA and 50 MIBC tumors profiled by DFCI/MSKCC predicted that 96% of patients had mutations detectable within the gene panel space [38,39] with a median of 5 mutations (non-silent and silent) detected per patient (**Fig 2B**).

We then tested the MRD panel for concordance of mutations detected in urine compared to tumor tissue. Five patients had paired urine and tumor tissue available for uCAPP-Seq using the MRD panel. Three patients had radical cystectomy tissue available, while 2 had transurethral resection of bladder tumor tissue from the time of diagnosis. Of note, none of these tissue samples had been affected by neoadjuvant chemotherapy. The concordance between urine- and tumor-derived mutations, determined in these 5 localized bladder cancer patients by uCAPP-Seq, was 85% (**S7 Table**).

### Non-silent mutations classify pathologic response

In our cohort, 38% (16/42) of patients achieved a pCR (*n* = 16), while 62% (26/42) had residual disease detected in their surgical sample (denoted as no pCR; *n* = 26). There were no significant differences between pCR and no pCR patients with regard to age, sex, ethnicity, smoking status, pretreatment tumor stage, receipt of neoadjuvant chemotherapy, or tumor histology (**S9 Table**). Furthermore, there was no significant difference in the concentration of urine cfDNA obtained between the 2 groups of patients.

We applied the MRD gene panel to the preoperative urine of these patients and detected a total of 52 non-silent mutations and 17 silent mutations (**S8 Table**). To have high confidence in our noninvasive variant calling, we only considered mutations detected in urine cfDNA with duplex support and with no read support in matched peripheral blood germline samples. Using the mutation with the highest variant allele fraction to represent the utDNA level, we compared the strength of association between pathologic response and utDNA detected from either non-silent or silent mutations. The ROC curve for non-silent mutations achieved a sensitivity of 81% and specificity of 81% (AUC = 0.78; 95% CI: 0.62 to 0.93), whereas the ROC curve for silent mutations achieved a sensitivity of only 12% (AUC = 0.53; 95% CI: 0.35 to 0.71) (**Fig 2C**). These results indicate that non-silent mutations classify pathologic response much more accurately than silent mutations, potentially because silent mutations are more likely to represent background field effect [18,46].

Consistent with increased background noise due to field effect, we observed that variant calling of non-silent, duplex-supported mutations within an expanded panel of 536 genes covering 387 kb of genomic space did not offer increased sensitivity and had inferior specificity (**S4 Fig**). The sensitivity only increased when variant calling was expanded to all duplex-supported mutations (silent or non-silent) within the expanded gene panel, but this also came at the expense of lower specificity. As a result, variant calling of non-silent, duplex-supported mutations within the driver gene-focused MRD panel yielded the best balance of sensitivity and specificity for classifying pathologic response in our cohort.

### No pCR is associated with higher utDNA levels than pCR

Focusing only on non-silent mutations in the MRD gene panel, we observed that patients with no pCR carried significantly more mutations (median of 2 mutations per patient) than patients with pCR (median of 0 mutations per patient). Urine cfDNA harbored a lower median number of mutations at the MRD time point than the aforementioned TCGA and DFCI/MSKCC studies [38,39], where tumor next-generation sequencing (NGS) was performed prior to any treatment. This decrease is consistent with prior ctDNA MRD studies in other solid tumor malignancies [23,25,47]. *TERT* and *TP53* were the most commonly altered genes detected in urine cfDNA across our 42 patient cohort (**Fig 2D**), consistent with the most commonly mutated genes in MIBC patients reported by TCGA and MSKCC [38,48]. *TERT* promoter and *TP53* mutations were detected in 24% (10/42) and 21% (9/42) of cases, respectively. uCAPP-Seq was also performed on 15 healthy adults to query the specificity of our MRD gene panel variant data. Only 2 mutations, one in the *PLEKHS1* intron and the other in *ATM*, were identified among these 15 healthy adults.

**Fig 3:**
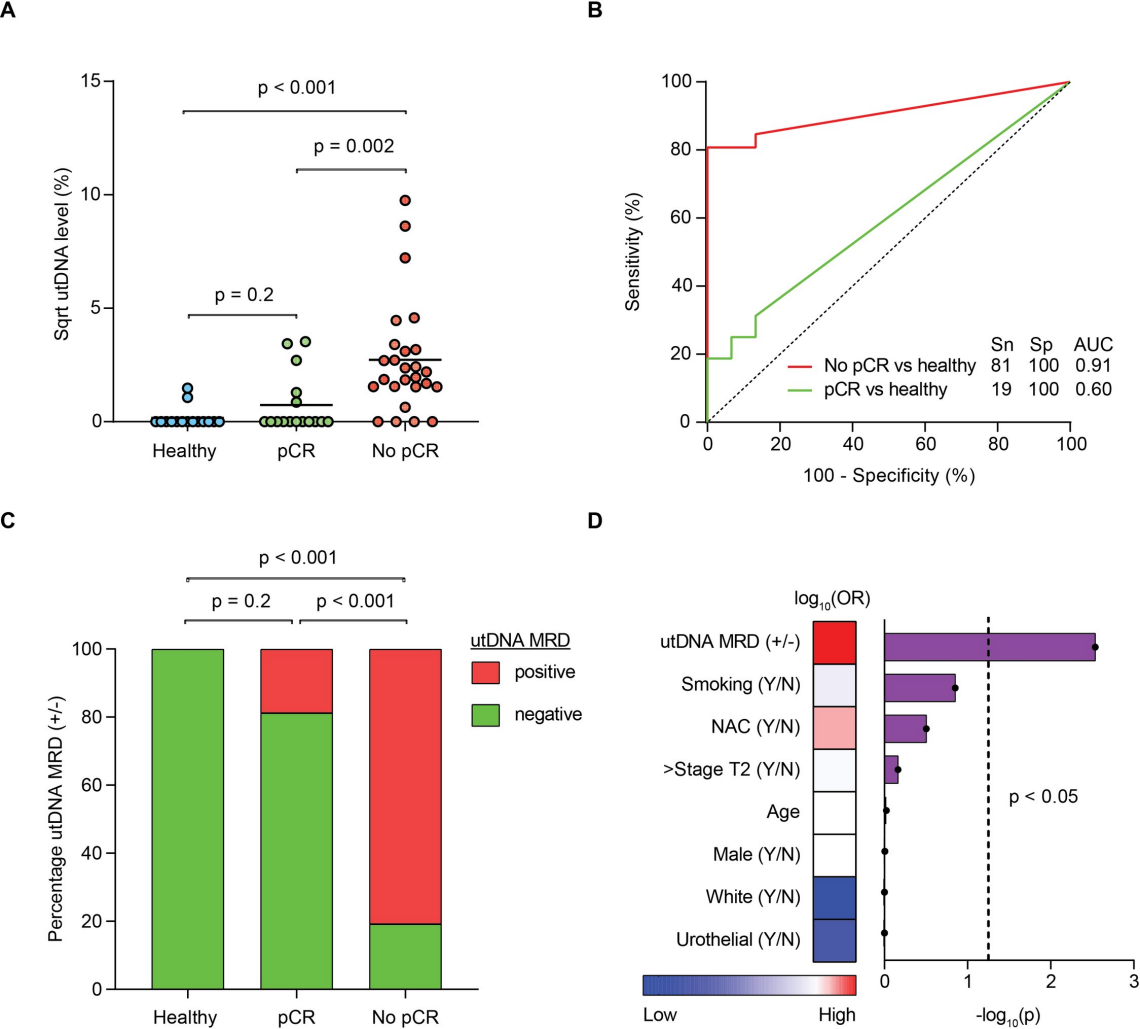
utDNA MRD analysis in patients with localized bladder cancer. **(A)** Scatter plot of utDNA MRD levels (highest non-silent VAF per patient at preoperative time point) after square root transformation for 3 groups: healthy adults, patients with pCR, and patients with no pCR. The *p*-values were calculated using Mann–Whitney U test with α of 0.017 after Bonferroni correction. **(B)** ROC analysis classifying localized bladder cancer patients (either pCR or no pCR) versus healthy adults by utDNA level. AUC and Youden index–optimized sensitivity and specificity values are shown. **(C)** Stacked bar plots showing the proportions of each group with positive or negative utDNA MRD detection, determined using the optimal utDNA level threshold (VAF: 2.3%) that classified patients with no pCR from healthy adults. The *p*-values were calculated using Fisher exact test with α of 0.017 after Bonferroni correction. **(D)** Multivariate logistic regression predicting the absence of pCR. The heatmap shows logarithm of the OR for each variable in the logistic regression. Each bar shows a negative logarithm of the *p*-value, with the dashed line denoting α of 0.05. AUC, area under the curve; MRD, minimal residual disease; OR, odds ratio; pCR, pathologic complete response; ROC, receiver operating characteristic; Sn, sensitivity; Sp, specificity; Sqrt, square root; utDNA, urine tumor DNA; VAF, variant allele fraction; Y/N, yes/no.

Moving forward in our analysis, we chose to represent a utDNA level for each patient based on the non-silent mutation with the highest variant allele fraction. Comparing utDNA levels between the groups, patients with no pCR had a significantly higher median utDNA level compared to patients with pCR (4.3% versus 0%, *p* = 0.002) and healthy adults (4.3% versus 0%, *p* < 0.001) (**Fig 3A**). In contrast, there was no difference in the median utDNA levels between healthy adults and patients with pCR (0% versus 0%, *p* = 0.2), implying that patients with pCR have similarly low biological background of urine variants as normal healthy adults. We then used variant data from the healthy adults to determine the optimal utDNA level threshold that classifies localized bladder cancer patients from healthy adults. Based on an ROC curve, this threshold was determined to be 2.3%, which classified patients with no pCR from healthy adults with a sensitivity of 81% and specificity of 100% (AUC = 0.91; 95% CI: 0.81 to 1.0) (**Fig 3B**). Therefore, patients with a utDNA level above or below this threshold were designated as utDNA MRD positive or negative, respectively.

The proportion of utDNA MRD–positive patients varied among patients with pCR, patients with no pCR, and healthy adults (**Fig 3C**). A total of 21 out of 26 (81%) patients with no pCR were utDNA MRD positive. Positive utDNA MRD detection was significantly associated with no pCR by Fisher exact test (*p* < 0.001) with a sensitivity of 81% and specificity of 81%. Positive predictive value (PPV) and negative predictive value (NPV) were 88% (21/24) and 72% (13/18), respectively. A multivariate logistic regression analysis was performed including 8 covariates to predict the absence of pCR: utDNA MRD detection, smoking status, administration of neoadjuvant chemotherapy, AJCC 8 tumor stage, age (continuous variable), sex, ethnicity, and histology (**Fig 3D**). This further corroborated utDNA MRD detection as the sole predictor of pathologic response (*p* = 0.003, odds ratio = 54.2), which established a clear separation between patients with pCR and patients with no pCR (**S10 Table**). Using utDNA MRD to predict pCR in a univariate logistic regression, we also applied leave-one-out cross-validation. Cross-validation revealed a highly significant accuracy of 81% (*p* = 0.007), which suggests that our prediction model generalizes well to independent data.

The association between pathologic response and utDNA remained highly significant when restricting our analysis to MIBC patients treated first with neoadjuvant chemotherapy. An ROC curve again showed that non-silent mutations (AUC = 0.84; 95% CI: 0.65 to 1.0) distinguish pCR from no pCR with higher accuracy than silent mutations (AUC = 0.55; 95% CI: 0.28 to 0.82) (**S5A Fig**). Moreover, patients with no pCR had a significantly higher utDNA level than patients with pCR (2.4% versus 0%, *p* = 0.006) (**S5B Fig**). Using the same utDNA threshold described above for the full cohort, 67% of patients with no pCR who received neoadjuvant chemotherapy were utDNA MRD positive, while 100% of patients with pCR who received neoadjuvant chemotherapy were utDNA MRD negative (**S5C Fig**). This difference in the proportion of patients designated as utDNA MRD positive between each pathologic response group was once again highly significant by Fisher exact test (*p* = 0.003). Notably, stratifying patients who received neoadjuvant chemotherapy by pathologic staging also showed a stepwise increase in detectable utDNA from ypT0N0 to ypTa/Tis N0 patients (**S5D Fig**). Thus, a utDNA threshold trained to distinguish ypT0N0 from ypTa/Tis N0 disease has the potential to be even more granular for detecting residual disease after neoadjuvant chemotherapy in MIBC patients.

### utDNA MRD as a prognostic biomarker

BC-1071 and BC-1206 are both MIBC patients who did not attain a pCR, and who, as expected, had positive utDNA MRD detection on preoperative urine sample collection. BC-1071 was a poor candidate for neoadjuvant chemotherapy and proceeded directly to radical cystectomy (**S6A Fig**). utDNA MRD was detected at a high level of 10.1% with nonsynonymous variation in the *KLF5* coding region and a UTR mutation in *TBC1D12*.

Nonetheless, the oncology team deferred adjuvant therapy as the patient was felt to be a poor candidate. Unfortunately, this patient developed metastasis to the liver 5.3 months after surgery and passed away shortly thereafter. Similarly, BC-1206 proceeded directly to radical cystectomy after oncologists considered this patient to be a suboptimal neoadjuvant chemotherapy candidate (**S6B Fig**). utDNA MRD was detected at a level of 2.8% with a stop–gain mutation in *TP53* and a nonsynonymous variation in the *ARID1A* coding region. Still, the patient elected to undergo active surveillance after surgery, but, unfortunately, developed progressive disease just 1.5 months later with peritoneal and bowel metastases and died 5.4 months thereafter.

**Fig 4:**
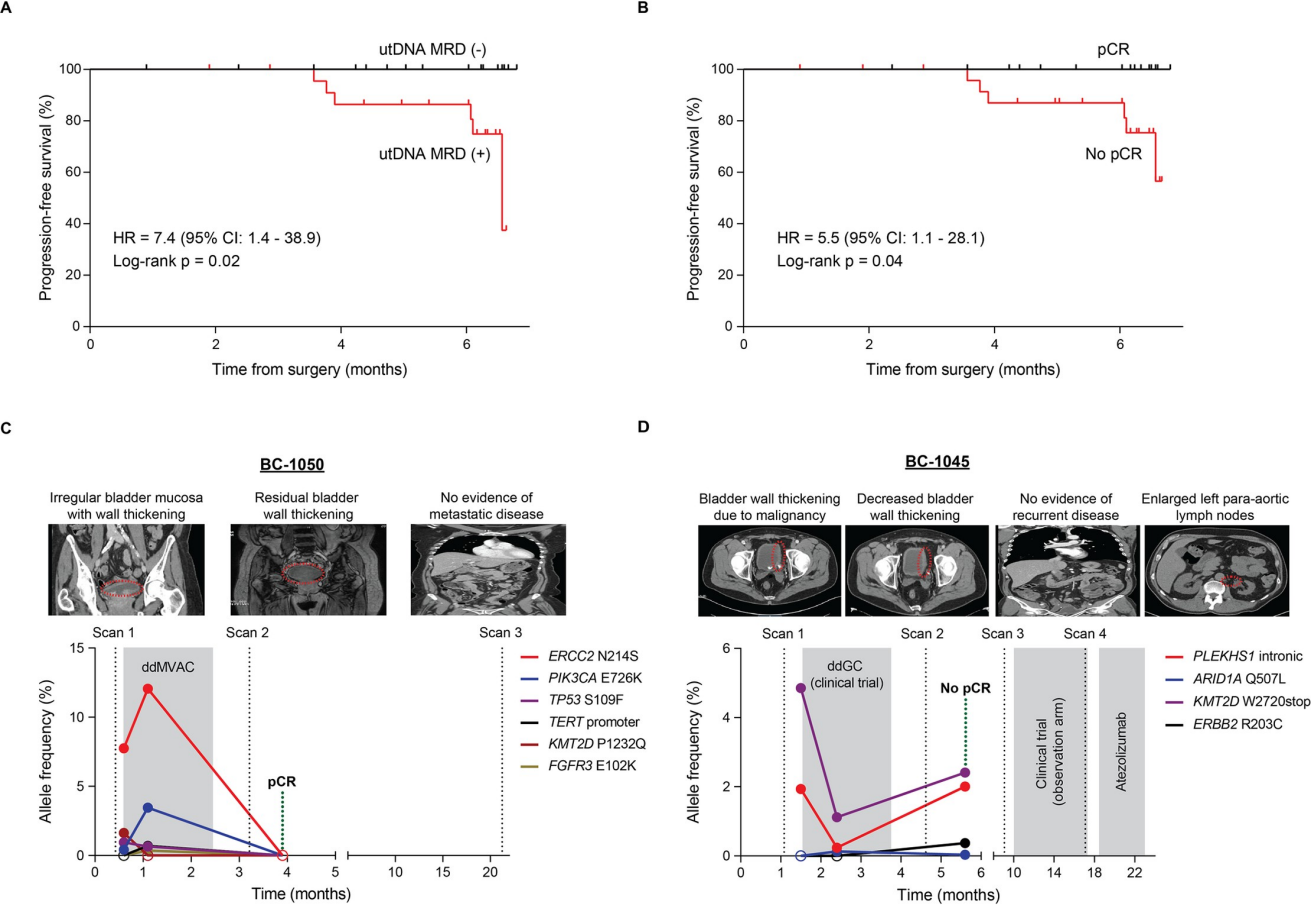
Preoperative utDNA MRD as an early prognostic biomarker in localized bladder cancer patients. **(A)** Kaplan–Meier analysis of PFS stratified by preoperative utDNA MRD detection and **(B)** pathologic response after radical cystectomy. utDNA MRD (+) patients had significantly worse PFS than utDNA MRD (−) patients (HR = 7.4; 95% CI: 1.4–38.9; *p* = 0.02). Patients with no pCR also had significantly worse PFS than patients with pCR (HR = 5.5; 95% CI: 1.1–28.1; *p* = 0.04). HRs and *p*-values were calculated using the Mantel–Haenszel and Mantel–Cox tests, respectively. **(C)** Serial urine cfDNA analysis for MIBC patient BC-1050. Imaging after neoadjuvant chemotherapy was equivocal with residual bladder wall thickening. utDNA levels increased during neoadjuvant chemotherapy with a COSMIC- and OncoKB-annotated nonsynonymous *ERCC2* mutation having the highest VAF. utDNA levels then decreased and became undetectable at the preoperative MRD time point, concordant with pCR on surgical pathology. The patient remained disease free on long-term follow-up. **(D)** Serial urine cfDNA analysis for MIBC patient BC-1045. Imaging after neoadjuvant chemotherapy noted decreased but persistent bladder wall thickening. utDNA levels decreased during neoadjuvant chemotherapy, with a *KMT2D* stop–gain mutation having the highest VAF. utDNA levels then increased at the preoperative MRD time point with evidence of 2 new emergent nonsynonymous mutations in *ERBB2* and *ARID1A*, not present earlier. Consistent with this positive utDNA MRD result, the patient had pathological nodal involvement despite achieving a pCR in the urothelium, and, less than a year later, developed progressive disease that prompted the start of treatment with atezolizumab. cfDNA, cell-free DNA; CI, confidence interval; COSMIC, Catalogue of Somatic Mutations in Cancer; HR, hazard ratio; MIBC, muscle-invasive bladder cancer; MRD, minimal residual disease; pCR, pathologic complete response; PFS, progression-free survival; utDNA, urine tumor DNA; VAF, variant allele fraction.

These clinical vignettes raised the question to us of how utDNA MRD detection compared to pathologic response in terms of predicting clinical outcomes, such as PFS and OS. We thus performed Kaplan–Meier analysis of patients in our cohort based on either utDNA MRD detection or pathologic response for PFS and OS. Six patients in our cohort recurred within 200 days with a median time to recurrence of 148 days. Notably, all 6 of these early-relapsed patients were utDNA MRD positive and exhibited significantly worse PFS compared to utDNA MRD–negative patients (HR = 7.4; 95% CI: 1.4 to 38.9; *p* = 0.02) (**Fig 4A**). This effect size was comparable to the PFS difference between patients with pCR compared to no pCR (HR = 5.5; 95% CI: 1.1 to 28.1; *p* = 0.04) (**Fig 4B**), suggesting that utDNA MRD detection is on par with pCR measured by a board-certified genitourinary pathologist in predicting PFS for up 6 months after surgery. After 6 months, utDNA MRD detection may potentially be superior to pathologic response, but more data are required to confirm this. Furthermore, utDNA MRD detection (HR = 5.0; 95% CI: 0.7 to 37.6; *p* = 0.12) (**S7A Fig**) and pathologic response (HR = 4.9; 95% CI: 0.6 to 37.2; *p* = 0.13) (**S7B Fig**) corresponded to differences in OS, but analysis of OS was not powered for statistical significance.

### Serial tracking of utDNA variants during neoadjuvant chemotherapy

We also correlated utDNA MRD detection with pathologic response for 2 patients (BC-1050 and BC-1045) who were treated with neoadjuvant chemotherapy and had multiple urine samples collected at different time points preceding radical cystectomy (**S11 Table**). We obtained serial urine samples from these 2 patients prior to starting neoadjuvant chemotherapy, during neoadjuvant chemotherapy, and on the day of radical cystectomy (MRD time point). All variants detected were monitored at each time point with CAPP-Seq using the previously described approach [20,21,28].

BC-1050 is a MIBC patient treated with 4 cycles of dose-dense methotrexate, vinblastine, doxorubicin, and cisplatin (**Fig 4C**). Surveillance imaging after neoadjuvant chemotherapy continued to denote minimal bladder wall thickening, which was equivocal for reflecting treatment response versus residual disease. utDNA MRD was not detected, however, suggesting a complete tumor response to neoadjuvant chemotherapy. The patient proceeded to radical cystectomy, where surgical pathology revealed pCR. The patient furthermore remained disease free on longer-term follow-up. Thus, both pCR and disease-free survival corroborated the negative utDNA MRD result.

Strikingly, additional utDNA analysis of serial urine samples preceding the MRD time point reflected changes in variant allele frequencies that were consistent with treatment response (**Fig 4C**). Prior to starting neoadjuvant chemotherapy, 4 non-silent mutations were detected with the highest being an *ERCC2* nonsynonymous driver mutation, which has been associated with increased response to chemotherapy [37,39]. Then, during chemotherapy (before starting the second cycle), half of these mutations, including this same *ERCC2* mutation, showed increased allele frequencies by an average of 5-fold relative to the initial measurement. Two new mutations that were previously undetected before chemotherapy also emerged at this time point during chemotherapy. However, these mutations all decreased significantly and became undetectable at the MRD time point, reflecting a robust response to neoadjuvant chemotherapy consistent with the pCR result. Thus, in retrospect, this patient represents an intriguing candidate for future clinical trials testing utDNA as a means to personalize the duration of neoadjuvant chemotherapy and potentially optimize candidacy for bladder-sparing treatment.

BC-1045 is another MIBC patient who was treated with 5 cycles of dose-dense gemcitabine and cisplatin on a clinical trial, and the sixth cycle was held due to neuropathy and tinnitus (**Fig 4D**). Surveillance imaging after chemotherapy noted a decrease, but not a complete disappearance, of baseline bladder wall thickening. Imaging also never revealed suspicion for nodal involvement. Preoperative utDNA MRD was detected, however, with the highest mutant allele frequency being in *KMT2D*, which harbored a stop–gain mutation.

The patient went on to receive a radical cystectomy, and notably pathologic assessment revealed no residual tumor in the bladder, suggesting a pCR in the urothelium (**Fig 4D**). Nodal disease was identified in the surgical specimen, however, which had been missed in the 2 prior diagnostic imaging studies. On subsequent surveillance, the patient developed metastatic left para-aortic lymphadenopathy within a year of surgery, which necessitated immune checkpoint blockade. In this challenging diagnostic case where preoperative imaging and pathologic analysis of the urothelium could have been misleading, utDNA MRD analysis was able to correctly identify nodal disease involvement and correlated with early disease progression on follow-up. Thus, the presence of utDNA in this patient may have represented plasma-derived transrenal cfDNA originating from disease outside the urothelium. This suggests that our assay has the potential to detect nodal or distant disease in the absence of intravesical disease.

For BC-1045, we also performed utDNA analysis of serial urine samples before and during neoadjuvant chemotherapy (**Fig 4D**). Shortly after diagnosis and prior to any neoadjuvant chemotherapy, the patient’s urine harbored non-silent mutations in *PLEKHS1* and *KMT2D*, present at variant allele frequencies of 1.9% and 4.9%, respectively. Interestingly, both of these mutations decreased during neoadjuvant chemotherapy with their allele frequencies going down by an average of 6.2-fold, consistent with the partial treatment response seen on imaging following chemotherapy. However, utDNA analysis at the preoperative MRD time point revealed a subsequent increase in the allele frequencies of these 2 mutations by an average of 5.2-fold, reflecting the development of treatment resistance, further highlighted by the emergence of newly detected mutations in *ARID1A* and *ERBB2* since chemotherapy was initiated. This patient represents a fascinating candidate for future clinical trials testing utDNA to personalize the regimentation of neoadjuvant and adjuvant systemic treatment based on serial utDNA analysis.

### utDNA MRD oncogenomics for treatment personalization

**Fig 5:**
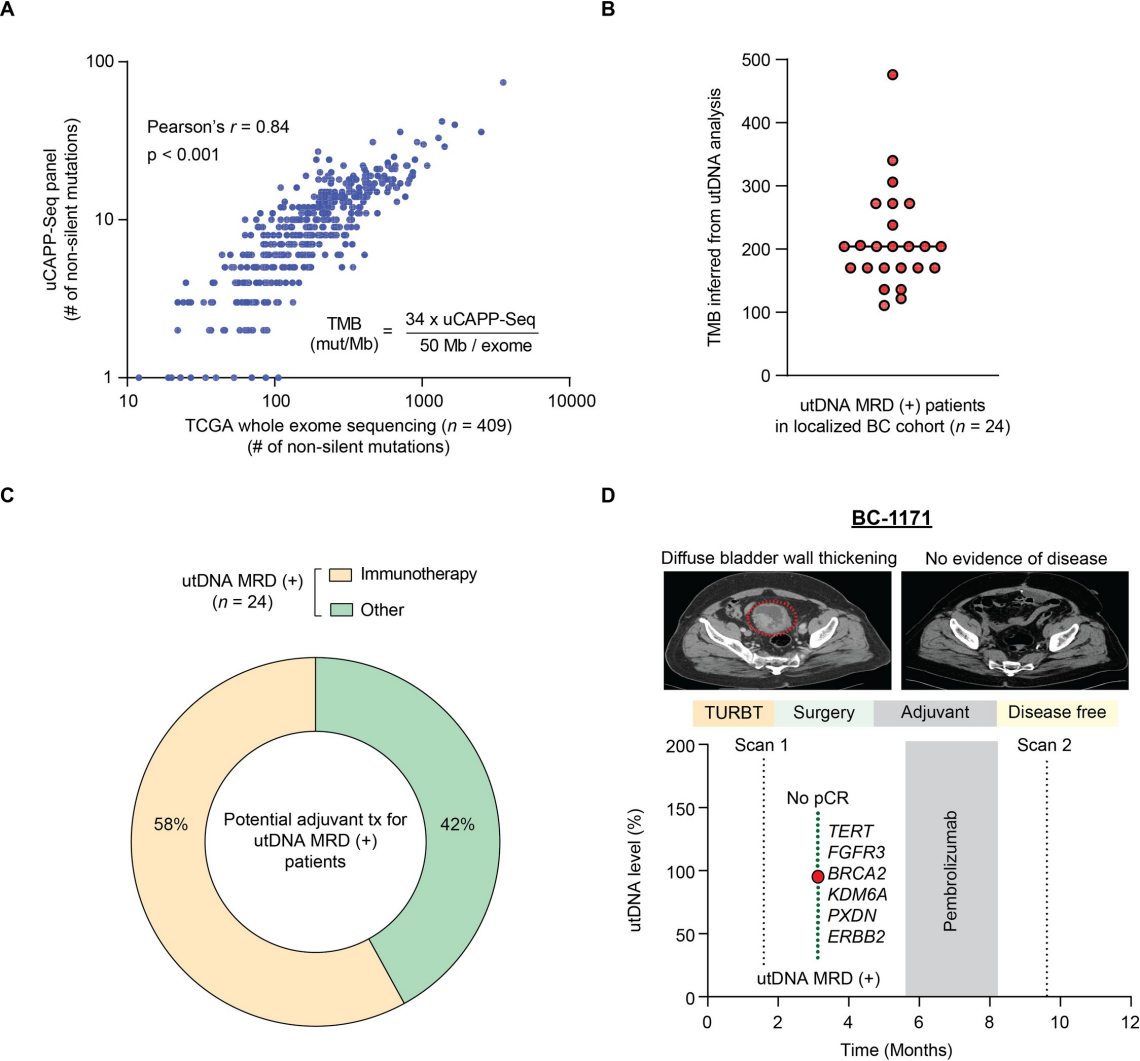
utDNA MRD as a biomarker for adjuvant treatment personalization. **(A)** Comparison of non-silent mutational load detected by whole exome sequencing and uCAPP-Seq for MIBC cases in TCGA (*n* = 409), using an expanded gene panel for assessing TMB (referred to as the TMB panel; **S3 Table**). Linear regression was performed (Pearson’s *r* = 0.84; equation shown) to interpolate exome-wide TMB based on the number of non-silent mutations detected by uCAPP-Seq. **(B)** Scatter plot depicting inferred TMB among patients with positive utDNA MRD in our cohort with the median indicated by a horizontal black line. **(C)** Potential adjuvant treatment strategies for utDNA MRD (+) patients based on urine-inferred TMB. **(D)** Vignette depicting MIBC patient BC-1171 who was utDNA MRD (+) at the preoperative time point, concordant with no pCR on surgical pathology with evidence of locally advanced pT3a disease (AJCC 8). Six non-silent mutations were detected in preoperative urine from this patient as indicated. Urine-inferred TMB was elevated at 204. This patient was randomized to pembrolizumab on a clinical trial and has shown no evidence of disease on long-term follow-up, consistent with our prediction for a patient with high inferred TMB. AJCC 8, American Joint Committee on Cancer 8th edition; MIBC, muscle-invasive bladder cancer; MRD, minimal residual disease; pCR, pathologic complete response; TCGA, The Cancer Genome Atlas; TMB, tumor mutational burden; TURBT, transurethral resection of bladder tumor; tx, treatment; uCAPP-Seq, urine Cancer Personalized Profiling by Deep Sequencing; utDNA, urine tumor DNA.

Although neoadjuvant immunotherapy has been approved for frontline administration in cisplatin-ineligible patients with metastatic bladder cancer, its use in the adjuvant setting is still being validated with several large clinical trials currently underway [49–51]. We and others have previously shown that TMB can be inferred from hybrid-capture cfDNA analysis in patients with non-small cell lung or colorectal cancer [23,47,52]. To determine if similar methodology can be applied to bladder cancer, we generated a linear regression model based on whole exome sequencing of 409 MIBC tumors in TCGA and the corresponding non-silent mutational burden detected by an expanded hybrid-capture panel for assessing TMB (**Fig 5A**, **S3 Table**) [38]. From this correlation (Pearson’s *r* = 0.84, *p* < 0.001), we derived an equation to infer exome-wide mutational burden from non-silent mutations in utDNA detected by our TMB panel. Using this equation to infer TMB among utDNA MRD–positive patients in our cohort (*n* = 24), the median TMB was 204 (range of 111 to 476) non-silent mutations per exome (**Fig 5B**, **S12 Table**).

High TMB is predictive of response to immune checkpoint blockade based on data from multiple studies in lung and bladder cancers [53,54]. A recent retrospective study of 139 patients with advanced, unresectable urothelial carcinoma receiving nivolumab on a clinical trial (CheckMate 275) revealed that TMB categorization by tertiles, with the highest TMB tertile defined as 170 missense mutations or more, was significantly associated with improved objective response rate (ORR), PFS, and OS [55]. Given the prognostic potential of this high TMB cutoff, we suggest that patients with an inferred TMB greater than 170 non-silent mutations per exome may be classified as high TMB. Based on this cutoff, we hypothesize that 58% of utDNA MRD–positive patients in our cohort with high urine–inferred TMB could potentially be identified as candidates for adjuvant immunotherapy (**Fig 5C**), thus facilitating the personalization of immune checkpoint blockade in the adjuvant setting for MIBC.

We also queried our cohort for potentially actionable mutations based on utDNA analysis, which revealed that 13% of utDNA MRD–positive patients also harbor mutations that have been shown to predict response to a drug in either bladder cancer or another solid tumor malignancy type [45] (**S8 Table**). Potentially actionable mutations that we identified include an *ERCC2* nonsynonymous mutation that is indexed in both the Catalogue of Somatic Mutations in Cancer (COSMIC) [56] and OncoKB [45] and correlates strongly with increased sensitivity to cisplatin-based chemotherapy. Other potentially actionable mutations we identified were nonsynonymous mutations in *PIK3CA* and *TSC1*, also annotated in both COSMIC [56] and OncoKB [45], and shown to be clinically targetable in breast cancer through PI3K inhibition [57,58] and in central nervous system cancers with mTOR inhibition [59], respectively.

The remaining 42% of utDNA MRD–positive patients in our cohort harbored mutations that were neither known to be actionable nor present at elevated burdens. We thus suggest that these MRD–positive patients might be appropriate candidates for other types of adjuvant therapy to ablate residual disease, such as chemotherapy or radiation therapy. Overall, the paradigm we describe in **Fig 5C** could thus form the basis for future clinical trials utilizing utDNA-based personalization of adjuvant treatment for MIBC patients harboring MRD.

In this regard, we identified 2 utDNA MRD–positive patients with high urine–inferred TMB in our cohort who received immunotherapy on a clinical trial. BC-1171 was randomized to adjuvant treatment with pembrolizumab after radical cystectomy (**Fig 5D**). This patient harbored an inferred TMB of 204 mutations per exome, thus classifying this patient as high TMB using our criteria. This patient has shown no evidence of recurrence or progression since surgery, which is consistent with our prediction based on our proposed paradigm. BC-1045, who was discussed previously (**Fig 4D**), was randomized to the observation arm of this same trial (versus pembrolizumab). The patient was diagnosed with disease progression 10.2 months following surgery and thus enrolled onto a different trial, where they are receiving treatment with single-agent atezolizumab. With an elevated urine-inferred TMB of 340 mutations per exome at the preoperative MRD time point, this patient has achieved a radiographic complete response in the involved lymph nodes on atezolizumab. It is tempting to speculate that this patient with high utDNA–inferred TMB may have averted disease relapse altogether if offered immunotherapy shortly after the MRD time point.

## Discussion

Here we present a cohort study that demonstrates the significant correlation between utDNA MRD detected by uCAPP-Seq prior to curative-intent radical cystectomy and pathologic response determined by analysis of the surgical specimen. We demonstrated this by utDNA analysis with a focused gene panel of non-silent mutations in consensus driver genes, which indicates that association with pathologic response is strongly linked to the detection of mutations that impact the phenotype of bladder cancer and are less likely to be confounded by background field effect [18,46]. As expected, the most commonly mutated genes detected in urine cfDNA in our cohort were *TERT* and *TP53*, consistent with the results of larger tumor sequencing studies [18,38,39]. Our approach also demonstrates an 85% level of concordance among mutations detected between tumor and utDNA for the subset of patients with tumor tissue available for analysis. Of note, our MRD detection approach only utilizes mutations with duplex support, which facilitates the low limit of detection necessary for MRD detection [21]. It also does not require prior sequencing of tumor tissue, which is practically useful and biologically can help avoid confounding of results due to geographic tumor heterogeneity [22].

Targeted NGS has previously been applied to detect early-stage bladder cancer in reasonably large cohorts using methods including UroSEEK [60] and uCAPP-Seq [28]. utMeMa, a methylation-based assessment of utDNA, has also shown feasibility in the MRD setting [61]. However, these previous urine-based sequencing approaches were either largely restricted to or exclusively studied in NMIBC and did not corroborate residual disease detection with a radical cystectomy specimen, which is currently the most accurate assessment of residual disease after standard of care treatment for MIBC. Moreover, there are several key differences in the biology and prognosis of NMIBC and MIBC, as the latter often arises via progression of urothelial dysplasia to muscle-invasive disease due to the accumulation of genomic alterations [62]. This is potentially related to an exacerbated field cancerization effect that may increase the risk of advanced disease and is associated with higher levels of nonspecific genomic alterations [63]. These distinctions require important technological advancements that optimize variant calling for MIBC.

In our study, we focused predominantly on MIBC with every patient being treated with radical cystectomy as part of their clinical management. Moreover, given that our cohort was predominantly composed of muscle-invasive patients, a high percentage received neoadjuvant chemotherapy. In this regard, we were able to apply state-of-the-art targeted deep sequencing using uCAPP-Seq technology to identify duplex-supported variants and analyze MRD in an important population not thoroughly assessed previously. Moreover, we correlated our findings with gold standard clinical outcomes including pathologic response, PFS, and OS.

Our data suggest that utDNA MRD detection can accurately predict pathologic response in MIBC patients. Predicting pathologic response in this fashion could enable personalization of treatment decisions for MIBC patients in the future. For example, utDNA MRD detection status could help inform bladder-sparing treatment, potentially enabling clinicians to forego radical cystectomy altogether in selected patients found to be MRD negative. Conversely, patients found to be utDNA MRD positive could be prioritized for further treatment such as targeted or immune therapy, which we suggest could be personalized based on the oncogenomic features learned from utDNA analysis. Notably, we did not detect any activating mutations in *FGFR3* in our cohort, which have been shown to predict responses to tyrosine kinase inhibitors in bladder cancer [64]. For example, erdafitinib is an *FGFR* inhibitor that is FDA approved for use in patients with locally advanced or metastatic bladder cancer [65]. Therefore, in addition to the mutations we highlighted in our cohort, utDNA could have the potential to enable personalized systemic treatment with erdafitinib for patients harboring *FGFR* mutations in the near future.

We additionally show through clinical vignettes that monitoring of utDNA before, during, and after neoadjuvant chemotherapy can be used to temporally monitor treatment response versus the emergence of resistance. Using this type of temporally high-resolution utDNA analysis, we envision that the strength and regimentation of neoadjuvant treatment could be further optimized, along with the consideration and timing of radical cystectomy. utDNA could also be used as an adjunct to other emerging modalities in this regard, such as VI-RADS, a newly developed MRI-based staging criteria [66], to more definitively predict treatment response and clinical outcomes in the future.

Limitations of our study include that utDNA analysis was performed considering only SNVs, which are the most predominant genomic alterations observed in bladder cancer [38,67], a cancer type with among the highest SNV burdens overall [68]. Importantly, since a false negative (i.e., no pCR falsely identified as utDNA MRD negative) could potentially result in a fatal outcome if used to guide clinical decision-making, our assay is not yet useful for routine clinical application. Additionally, the findings we present here will need to be independently validated before clinical implementation. As the tumor genomic characterization of MIBC broadens in the future, the sensitivity of our assay should improve. Future work to improve the sensitivity of residual disease detection could also explore the combination of utDNA analysis with clinical evaluation (i.e., cystoscopy), as well as incorporating other genomic alterations into our method (e.g., copy number variations, insertions/deletions, and fusions).

We also note that all utDNA testing in this study was performed prior to radical cystectomy, which enabled us to assess the ability of utDNA MRD to predict pathologic response. However, utDNA analysis at this time point makes it challenging to assess potential subsequent prognostic implications, since the risk of disease progression or death is typically diminished by curative-intent surgical intervention [2]. As a result, a utDNA MRD–positive patient at elevated risk of disease progression could have their MRD fully addressed via surgery, and, thus, could remain disease free in our Kaplan–Meier analysis. Even with this limitation, however, we observed a significant difference in PFS and likely a difference in OS. An ongoing prospective clinical trial utilizing a multi-institutional cohort through the Alliance study (NCT03609216) will aim to further validate the ability of utDNA to predict complete response and survival outcomes. Future work may also include studying a cohort of healthy smokers in order to examine if utDNA can be used as a screening tool for bladder cancer.

Finally, it has been shown that mutations in DNA damage repair genes, such as *ERCC2*, are predictive of chemotherapeutic response [37,39]. This is consistent with our observation of an overall decrease in variant allele frequencies in serial urine samples collected during and after neoadjuvant chemotherapy for patient BC-1050, who harbored a nonsynonymous *ERCC2* mutation. Still, we were unable to assess the association between DDR mutations and chemotherapeutic response in our cohort as a whole because these mutations at the MRD time point are mostly undetectable after chemotherapy is complete, as was the case for BC-1050. To increase the number of mutations detected in DNA damage repair genes, we would need to increase our study power by enrolling more patients or preferably collect and analyze more urine samples prior to neoadjuvant chemotherapy.

In conclusion, we have shown that utDNA MRD detection with uCAPP-Seq classifies pathologic response with high sensitivity and specificity in a cohort comprised predominantly of MIBC. Our approach has the potential to facilitate more personalized treatment interventions for MIBC in the future, such as curative-intent bladder-sparing approaches, adjuvant treatment with immune checkpoint blockade, and targeted systemic therapy administration. To establish clinical utility, these utDNA-guided interventions will need to be assessed in prospective clinical trials.

## Supporting information

S1 Checklist STROBE Statement—Checklist of items that should be included in reports of cohort studies. STROBE, Strengthening the Reporting of Observational Studies in Epidemiology.
(DOCX)

S1 Fig EDTA prevents urine cfDNA degradation. Technical experiment to evaluate the stability of urine cfDNA with and without the addition of EDTA. Pooled urine from a healthy volunteer was stored for 7 days with versus without 10 mM EDTA at room temperature. Urine cfDNA was extracted at days 0, 1, 3, and 7 after commencing storage. The effect of EDTA on urine cfDNA degradation was evaluated based on the percentage of DNA in the 70–450 bp size range on Bioanalyzer eletropherogram. The data presented here are mean ± SEM of 2 replicates per time point. The *p*-value was calculated by unpaired Student *t* test at the last time point. bp, base pair; cfDNA, cell-free DNA; SEM, standard error of the mean.
(TIF)

S2 Fig Schema for comparing in vitro size selection against acoustic fragmentation of urine cfDNA. Representative electropherograms are also shown, all from the same MIBC patient’s urine sample. bp, base pair; cfDNA, cell-free DNA; MIBC, muscle-invasive bladder cancer; mM, millimolar; uCAPP-Seq, urine Cancer Personalized Profiling by Deep Sequencing.
(TIF)

S3 Fig Comparison of acoustic whole urine cfDNA fragmentation to in vitro size selection of 70–450 bp cfDNA fragments. **(A)** Median deduplicated sequencing depths, **(B)** Number of non-silent mutations, **(C)** Mutant allele fraction levels in 4 MIBC urine cfDNA samples that underwent in vitro size selection or acoustic DNA fragmentation (see **S2 Fig** for schema). Data values are depicted as points, column heights represent averages, and error bars represent standard error of the mean. VAF comparisons in (C) are also shown with connecting lines. The *p*-values were calculated using the paired Student *t* test method. bp, base pair; cfDNA, cell-free DNA; MIBC, muscle-invasive bladder cancer; VAF, variant allele fraction.
(TIF)

S4 Fig ROC analysis classifying pCR versus no pCR with different variant calling criteria. The red curve shows variant calling of non-silent, duplex-supported mutations in the MRD panel. The blue curve shows variant calling of non-silent, duplex-supported mutations in the TMB panel. The green curve shows variant calling of all mutations (silent or non-silent) with duplex support in the TMB panel. The AUC, as well as the optimal sensitivity and specificity values corresponding to Youden J statistic, is shown. AUC, area under the curve; MRD, minimal residual disease; pCR, pathologic complete response; ROC, receiver operating characteristic; Sn, sensitivity; Sp, specificity; TMB, tumor mutational burden.
(TIF)

S5 Fig utDNA MRD analysis in MIBC patients treated with neoadjuvant chemotherapy. **(A)** ROC analysis classifying MIBC patients (either pCR or no pCR) who received neoadjuvant chemotherapy before radical cystectomy based on non-silent versus silent mutations detected in preoperative urine cfDNA. The AUC, as well as the optimal sensitivity and specificity values corresponding to Youden J statistic, is shown. **(B)** Scatter plot of utDNA levels (highest non-silent VAF per patient at the preoperative time point) after square root transformation in patients who achieved pCR versus those with no pCR following neoadjuvant chemotherapy. The *p*-value was calculated using Mann–Whitney U test. **(C)** Stacked bar plots showing proportions of each group with positive or negative utDNA MRD detection after neoadjuvant chemotherapy, determined by the optimal utDNA threshold (VAF: 2.3%) that classified patients with no pCR from healthy adults in the full patient cohort as described in the Methods. The *p*-value was calculated using Fisher exact test. **(D)** Scatter plot of utDNA levels after square root transformation in patients who received neoadjuvant chemotherapy, grouped by pathologic stage. Column heights depict mean values, and error bars represent the standard error of the mean. The Kruskal–Wallis H statistic and *p*-value are shown in the upper left. There were no patients with ypT1 disease. AUC, area under the curve; cfDNA, cell-free DNA; MIBC, muscle-invasive bladder cancer; MRD, minimal residual disease; pCR, pathologic complete response; ROC, receiver operating characteristic; Sn, sensitivity; Sp, specificity; Sqrt, square root; utDNA, urine tumor DNA; VAF, variant allele fraction.
(TIF)

S6 Fig Correlation of utDNA MRD detection with pathologic nonresponse in 2 MIBC patients. **(A)** Clinical vignette of MIBC patient BC-1071 who was utDNA MRD (+) at the preoperative time point with a utDNA level of 10.1%, which was concordant with no pCR on surgical pathology. The patient progressed 160 days later and died shortly thereafter. **(B)** Clinical vignette of MIBC patient BC-1206 who was utDNA MRD (+) at the preoperative time point with a utDNA level of 2.8%, which was concordant with no pCR on surgical pathology. The patient progressed at 46 days after radical cystectomy, received chemoradiation to treat the recurrence, but, nonetheless, died at 164 days. MIBC, muscle-invasive bladder cancer; MRD, minimal residual disease; pCR, pathologic complete response; TURBT, transurethral resection of bladder tumor; utDNA, urine tumor DNA.
(TIF)

S7 Fig Correlation of utDNA MRD detection and pathologic response with OS. OS analysis according to utDNA MRD detection at the preoperative time point **(A)** and pathologic response assessed in the radical cystectomy specimen **(B)**. HRs and *p*-values were calculated using the Mantel–Haenszel and Mantel–Cox methods, respectively. CI, confidence interval; HR, hazard ratio; MRD, minimal residual disease; OS, overall survival; pCR, pathologic complete response, utDNA, urine tumor DNA.
(TIF)

S1 Table Patient-level characteristics in the bladder cancer cohort. (XLSX)

S2 Table Genes fully or partially covered in the MRD panel. MRD, minimal residual disease.
(XLSX)

S3 Table Genes fully or partially covered in the TMB panel. TMB, tumor mutational burden.
(XLSX)

S4 Table Sequencing metrics of urine cfDNA for all patients. cfDNA, cell-free DNA.
(XLSX)

S5 Table Sequencing metrics of urine cfDNA in healthy adults. cfDNA, cell-free DNA.
(XLSX)

S6 Table Tumor tissue and germline DNA sequencing metrics. (XLSX)

S7 Table Mutational concordance between tumor tissue and urine cfDNA. cfDNA, cell-free DNA.
(XLSX)

S8 Table Mutations detected at the preoperative MRD time point in urine using the MRD panel. MRD, minimal residual disease.
(XLSX)

S9 Table Characteristics of patients according to pathologic response at surgery. (XLSX)

S10 Table Multivariate logistic regression to predict the absence of pathologic complete response. (XLSX)

S11 Table Serial monitoring of utDNA on neoadjuvant chemotherapy. utDNA, urine tumor DNA.
(XLSX)

S12 Table Urine-inferred tumor mutational burden for utDNA MRD positive patients. MRD, minimal residual disease; utDNA, urine tumor DNA.
(XLSX)

## Abbreviations

AJCC: American Joint Committee on Cancer
BCG: Bacillus Calmette–Guérin
CAPP-Seq: Cancer Personalized Profiling by Deep Sequencing
cfDNA: cell-free DNA
COSMIC: Catalogue of Somatic Mutations in Cancer
ctDNA: circulating tumor DNA
DDR: DNA damage response
DFCI/MSKCC: Dana-Farber Cancer Institute and Memorial Sloan Kettering Cancer Center
FDA: Food and Drug Administration
gnomAD: Genome Aggregation Database
iDES: integrated digital error suppression
IRB: Institutional Review Board
MIBC: muscle-invasive bladder cancer
MRD: minimal residual disease
NGS: next-generation sequencing
NMIBC: non–muscle-invasive bladder cancer
NPV: negative predictive value
ORR: objective response rate
OS: overall survival
pCR: pathologic complete response
PFS: progression-free survival
PPV: positive predictive value
ROC: receiver operating characteristic
SNV: single-nucleotide variant
STROBE: Strengthening the Reporting of Observational Studies in Epidemiology
TCGA: The Cancer Genome Atlas
TMB: tumor mutational burden
uCAPP-Seq: urine Cancer Personalized Profiling by Deep Sequencing
utDNA: urine tumor DNA

## References

[1] Siegel RL, Miller KD, Jemal A. Cancer statistics, 2020. CA Cancer J Clin. 2020;70(1):7–30. Epub 2020/01/09. doi: 10.3322/caac.21590 . 31912902

[2] Flaig TW, Spiess PE, Agarwal N, Bangs R, Boorjian SA, Buyyounouski MK, et al. Bladder Cancer, Version 3.2020, NCCN Clinical Practice Guidelines in Oncology. J Natl Compr Canc Netw. 2020;18(3):329–54. Epub 2020/03/07. doi: 10.6004/jnccn.2020.0011 . 32135513

[3] Kirkali Z, Chan T, Manoharan M, Algaba F, Busch C, Cheng L, et al. Bladder cancer: epidemiology, staging and grading, and diagnosis. Urology. 2005;66(6 Suppl 1):4–34. Epub 2006/01/10. doi: 10.1016/j.urology.2005.07.062 . 16399414

[4] Sylvester RJ, van der Meijden AP, Oosterlinck W, Witjes JA, Bouffioux C, Denis L, et al. Predicting recurrence and progression in individual patients with stage Ta T1 bladder cancer using EORTC risk tables: a combined analysis of 2596 patients from seven EORTC trials. Eur Urol. 2006;49(3):466–5; discussion 75–7. Epub 2006/01/31. doi: 10.1016/j.eururo.2005.12.031 . 16442208

[5] Sievert KD, Amend B, Nagele U, Schilling D, Bedke J, Horstmann M, et al. Economic aspects of bladder cancer: what are the benefits and costs? World J Urol. 2009;27(3):295–300. Epub 2009/03/10. doi: 10.1007/s00345-009-0395-z ; PubMed Central PMCID: PMC2694315. 19271220 PMC2694315

[6] Yeung C, Dinh T, Lee J. The health economics of bladder cancer: an updated review of the published literature. Pharmacoeconomics. 2014;32(11):1093–104. Epub 2014/07/25. doi: 10.1007/s40273-014-0194-2 . 25056838

[7] Cumberbatch MGK, Jubber I, Black PC, Esperto F, Figueroa JD, Kamat AM, et al. Epidemiology of Bladder Cancer: A Systematic Review and Contemporary Update of Risk Factors in 2018. Eur Urol. 2018;74(6):784–95. Epub 2018/10/01. doi: 10.1016/j.eururo.2018.09.001 . 30268659

[8] Mossanen M, Krasnow RE, Zlatev DV, Tan WS, Preston MA, Trinh QD, et al. Examining the relationship between complications and perioperative mortality following radical cystectomy: a population-based analysis. BJU Int. 2019;124(1):40–6. Epub 2018/12/01. doi: 10.1111/bju.14636 . 30499636

[9] Shabsigh A, Korets R, Vora KC, Brooks CM, Cronin AM, Savage C, et al. Defining early morbidity of radical cystectomy for patients with bladder cancer using a standardized reporting methodology. Eur Urol. 2009;55(1):164–74. Epub 2008/08/05. doi: 10.1016/j.eururo.2008.07.031 . 18675501

[10] Grossman HB, Natale RB, Tangen CM, Speights VO, Vogelzang NJ, Trump DL, et al. Neoadjuvant chemotherapy plus cystectomy compared with cystectomy alone for locally advanced bladder cancer. N Engl J Med. 2003;349(9):859–66. Epub 2003/08/29. doi: 10.1056/NEJMoa022148 . 12944571

[11] Vale CL. Neoadjuvant Chemotherapy in Invasive Bladder Cancer: Update of a Systematic Review and Meta-Analysis of Individual Patient Data: Advanced Bladder Cancer (ABC) Meta-analysis Collaboration. Eur Urol. 2005;48 (2):202–6. doi: 10.1016/j.eururo.2005.04.006 15939524

[12] Petrelli F, Coinu A, Cabiddu M, Ghilardi M, Vavassori I, Barni S. Correlation of pathologic complete response with survival after neoadjuvant chemotherapy in bladder cancer treated with cystectomy: a meta-analysis. Eur Urol. 2014;65(2):350–7. Epub 2013/07/16. doi: 10.1016/j.eururo.2013.06.049 . 23849998

[13] Iyer G, Balar AV, Milowsky MI, Bochner BH, Dalbagni G, Donat SM, et al. Multicenter Prospective Phase II Trial of Neoadjuvant Dose-Dense Gemcitabine Plus Cisplatin in Patients With Muscle-Invasive Bladder Cancer. J Clin Oncol. 2018;36(19):1949–56. Epub 2018/05/10. doi: 10.1200/JCO.2017.75.0158 ; PubMed Central PMCID: PMC6049398. 29742009 PMC6049398

[14] Li Q, Damish AW, Frazier Z, Liu D, Reznichenko E, Kamburov A, et al. ERCC2 Helicase Domain Mutations Confer Nucleotide Excision Repair Deficiency and Drive Cisplatin Sensitivity in Muscle-Invasive Bladder Cancer. Clin Cancer Res. 2019;25(3):977–88. Epub 2018/07/08. doi: 10.1158/1078-0432.CCR-18-1001 ; PubMed Central PMCID: PMC6434536. 29980530 PMC6434536

[15] Mazza P, Moran GW, Li G, Robins DJ, Matulay JT, Herr HW, et al. Conservative Management Following Complete Clinical Response to Neoadjuvant Chemotherapy of Muscle Invasive Bladder Cancer: Contemporary Outcomes of a Multi-Institutional Cohort Study. J Urol. 2018;200(5):1005–13. Epub 2018/05/23. doi: 10.1016/j.juro.2018.05.078 ; PubMed Central PMCID: PMC7543664. 29787740 PMC7543664

[16] Kukreja JB, Porten S, Golla V, Ho PL, Noguera-Gonzalez G, Navai N, et al. Absence of Tumor on Repeat Transurethral Resection of Bladder Tumor Does Not Predict Final Pathologic T0 Stage in Bladder Cancer Treated with Radical Cystectomy. Eur Urol Focus. 2018;4(5):720–4. Epub 2017/07/30. doi: 10.1016/j.euf.2016.12.005 . 28753837

[17] Becker REN, Meyer AR, Brant A, Reese AC, Biles MJ, Harris KT, et al. Clinical Restaging and Tumor Sequencing are Inaccurate Indicators of Response to Neoadjuvant Chemotherapy for Muscle-invasive Bladder Cancer. Eur Urol. 2020. Epub 2020/08/21. doi: 10.1016/j.eururo.2020.07.016. 32814637

[18] Chaudhuri AA, Pellini B, Pejovic N, Chauhan PS, Harris PK, Szymanski JJ, et al. Emerging Roles of Urine-Based Tumor DNA Analysis in Bladder Cancer Management. JCO Precis Oncol. 2020;4. Epub 2020/09/15. doi: 10.1200/PO.20.00060; PubMed Central PMCID: PMC7448529. 32923907 PMC7448529

[19] Vandekerkhove G, Lavoie J-M, Annala M, Murtha AJ, Sundahl N, Walz S, et al. Plasma ctDNA is a tumor tissue surrogate and enables clinical-genomic stratification of metastatic bladder cancer. Nat Commun. 2021;12 (1):184. doi: 10.1038/s41467-020-20493-6 33420073 PMC7794518

[20] Newman AM, Bratman SV, To J, Wynne JF, Eclov NC, Modlin LA, et al. An ultrasensitive method for quantitating circulating tumor DNA with broad patient coverage. Nat Med. 2014;20(5):548–54. Epub 2014/04/08. doi: 10.1038/nm.3519 ; PubMed Central PMCID: PMC4016134. 24705333 PMC4016134

[21] Newman AM, Lovejoy AF, Klass DM, Kurtz DM, Chabon JJ, Scherer F, et al. Integrated digital error suppression for improved detection of circulating tumor DNA. Nat Biotechnol. 2016;34(5):547–55. Epub 2016/03/29. doi: 10.1038/nbt.3520 ; PubMed Central PMCID: PMC4907374. 27018799 PMC4907374

[22] Chin RI, Chen K, Usmani A, Chua C, Harris PK, Binkley MS, et al. Detection of Solid Tumor Molecular Residual Disease (MRD) Using Circulating Tumor DNA (ctDNA). Mol Diagn Ther. 2019;23(3):311–31. Epub 2019/04/04. doi: 10.1007/s40291-019-00390-5 ; PubMed Central PMCID: PMC6561896. 30941670 PMC6561896

[23] Chaudhuri AA, Chabon JJ, Lovejoy AF, Newman AM, Stehr H, Azad TD, et al. Early Detection of Molecular Residual Disease in Localized Lung Cancer by Circulating Tumor DNA Profiling. Cancer Discov. 2017;7(12):1394–403. Epub 2017/09/14. doi: 10.1158/2159-8290.CD-17-0716 ; PubMed Central PMCID: PMC5895851. 28899864 PMC5895851

[24] Abbosh C, Birkbak NJ, Swanton C. Early stage NSCLC—challenges to implementing ctDNA-based screening and MRD detection. Nat Rev Clin Oncol. 2018;15(9):577–86. Epub 2018/07/04. doi: 10.1038/s41571-018-0058-3 . 29968853

[25] Azad TD, Chaudhuri AA, Fang P, Qiao Y, Esfahani MS, Chabon JJ, et al. Circulating Tumor DNA Analysis for Detection of Minimal Residual Disease After Chemoradiotherapy for Localized Esophageal Cancer. Gastroenterology. 2020;158(3):494–505 e6. Epub 2019/11/13. doi: 10.1053/j.gastro.2019.10.039 ; PubMed Central PMCID: PMC7010551. 31711920 PMC7010551

[26] Scherer F, Kurtz DM, Newman AM, Stehr H, Craig AF, Esfahani MS, et al. Distinct biological subtypes and patterns of genome evolution in lymphoma revealed by circulating tumor DNA. Sci Transl Med. 2016;8(364):364ra155. Epub 2016/11/11. doi: 10.1126/scitranslmed.aai8545; PubMed Central PMCID: PMC5490494. 27831904 PMC5490494

[27] Przybyl J, Chabon JJ, Spans L, Ganjoo KN, Vennam S, Newman AM, et al. Combination Approach for Detecting Different Types of Alterations in Circulating Tumor DNA in Leiomyosarcoma. Clin Cancer Res. 2018;24(11):2688–99. Epub 2018/02/22. doi: 10.1158/1078-0432.CCR-17-3704 ; PubMed Central PMCID: PMC5984700. 29463554 PMC5984700

[28] Dudley JC, Schroers-Martin J, Lazzareschi DV, Shi WY, Chen SB, Esfahani MS, et al. Detection and Surveillance of Bladder Cancer Using Urine Tumor DNA. Cancer Discov. 2019;9(4):500–9. Epub 2018/12/24. doi: 10.1158/2159-8290.CD-18-0825 ; PubMed Central PMCID: PMC6467650. 30578357 PMC6467650

[29] Lui YY, Woo KS, Wang AY, Yeung CK, Li PK, Chau E, et al. Origin of plasma cell-free DNA after solid organ transplantation. Clin Chem. 2003;49(3):495–6. Epub 2003/02/26. doi: 10.1373/49.3.495 . 12600963

[30] Oellerich M, Shipkova M, Asendorf T, Walson PD, Schauerte V, Mettenmeyer N, et al. Absolute quantification of donor-derived cell-free DNA as a marker of rejection and graft injury in kidney transplantation: Results from a prospective observational study. Am J Transplant. 2019;19(11):3087–99. Epub 2019/05/08. doi: 10.1111/ajt.15416 ; PubMed Central PMCID: PMC6899936. 31062511 PMC6899936

[31] Alliance for Clinical Trials in Oncology. Dose Dense Gemcitabine and Cisplatin Without Cystectomy for Patients With Muscle Invasive Bladder Urothelial Cancer and Select Genetic Alterations. [ClinicalTrials.gov identifier NCT 03609216]. National Institutes of Health, ClinicalTrials.gov. Available from: https://clinicaltrials.gov/ct2/show/NCT03609216. Accessed 2021 Jan 11.

[32] Pathological Complete Response in Neoadjuvant Treatment of High-Risk Early-Stage Breast Cancer: Use as an Endpoint to Support Accelerated Approval. FDA-2012-D-0432. Center for Drug Evaluation and Research; July 2020.

[33] Amin MBES, Greene F, Byrd DR, Brookland RK, Washington MK, et al. AJCC Cancer Staging Manual: Springer International Publishing; 2017. XVII:1032 p.

[34] Dang HX, Chauhan PS, Ellis H, Feng W, Harris PK, Smith G, et al. Cell-free DNA alterations in the AR enhancer and locus predict resistance to AR-directed therapy in patients with metastatic prostate cancer. JCO Precis Oncol. 2020;4:680–713. Epub 2020/09/10. doi: 10.1200/po.20.00047 ; PubMed Central PMCID: PMC7446541. 32903952 PMC7446541

[35] Shekhtman EM, Anne K, Melkonyan HS, Robbins DJ, Warsof SL, Umansky SR. Optimization of transrenal DNA analysis: detection of fetal DNA in maternal urine. Clin Chem. 2009;55(4):723–9. Epub 2009/02/03. doi: 10.1373/clinchem.2008.113050 . 19181739

[36] Jeeta RR, Gordon NS, Baxter L, Goel A, Noyvert B, Ott S, et al. Non-Coding Mutations in Urothelial Bladder Cancer: Biological and Clinical Relevance and Potential Utility as Biomarkers. Bladder Cancer. 2019;5:263–72. doi: 10.3233/BLC-190251 PMC7390591 32793790

[37] Liu D, Plimack ER, Hoffman-Censits J, Garraway LA, Bellmunt J, Van Allen E, et al. Clinical Validation of Chemotherapy Response Biomarker ERCC2 in Muscle-Invasive Urothelial Bladder Carcinoma. JAMA Oncol. 2016;2(8):1094–6. Epub 2016/06/17. doi: 10.1001/jamaoncol.2016.1056 ; PubMed Central PMCID: PMC5515075. 27310333 PMC5515075

[38] Robertson AG, Kim J, Al-Ahmadie H, Bellmunt J, Guo G, Cherniack AD, et al. Comprehensive Molecular Characterization of Muscle-Invasive Bladder Cancer. Cell. 2018;174(4):1033. Epub 2018/08/11. doi: 10.1016/j.cell.2018.07.036; PubMed Central PMCID: PMC6297116. 30096301 PMC6297116

[39] Van Allen EM, Mouw KW, Kim P, Iyer G, Wagle N, Al-Ahmadie H, et al. Somatic ERCC2 mutations correlate with cisplatin sensitivity in muscle-invasive urothelial carcinoma. Cancer Discov. 2014;4(10):1140–53. Epub 2014/08/07. doi: 10.1158/2159-8290.CD-14-0623 ; PubMed Central PMCID: PMC4238969. 25096233 PMC4238969

[40] Weinstein JN, Akbani R, Broom BM, Wang W, Verhaak RGW, McConkey D, et al. Comprehensive molecular characterization of urothelial bladder carcinoma. Nature. 2014;507 (7492):315–22. doi: 10.1038/nature12965 24476821 PMC3962515

[41] Karczewski KJ, Francioli LC, Tiao G, Cummings BB, Alfoldi J, Wang Q, et al. The mutational constraint spectrum quantified from variation in 141,456 humans. Nature. 2020;581(7809):434–43. Epub 2020/05/29. doi: 10.1038/s41586-020-2308-7 ; PubMed Central PMCID: PMC7334197. 32461654 PMC7334197

[42] Wang K, Li M, Hakonarson H. ANNOVAR: functional annotation of genetic variants from high-throughput sequencing data. Nucleic Acids Res. 2010;38(16):e164. Epub 2010/07/06. doi: 10.1093/nar/gkq603; PubMed Central PMCID: PMC2938201. 20601685 PMC2938201

[43] Abbosh PH, Plimack ER. Molecular and Clinical Insights into the Role and Significance of Mutated DNA Repair Genes in Bladder Cancer. Bladder Cancer. 2018;4(1):9–18. Epub 2018/02/13. doi: 10.3233/BLC-170129 ; PubMed Central PMCID: PMC5798524. 29430503 PMC5798524

[44] Cerami E, Gao J, Dogrusoz U, Gross BE, Sumer SO, Aksoy BA, et al. The cBio cancer genomics portal: an open platform for exploring multidimensional cancer genomics data. Cancer Discov. 2012;2(5):401–4. Epub 2012/05/17. doi: 10.1158/2159-8290.CD-12-0095 ; PubMed Central PMCID: PMC3956037. 22588877 PMC3956037

[45] Chakravarty D, Gao J, Phillips SM, Kundra R, Zhang H, Wang J, et al. OncoKB: A Precision Oncology Knowledge Base. JCO Precis Oncol. 2017;2017. doi: 10.1200/PO.17.00011; PubMed Central PMCID: PMC5586540. 28890946 PMC5586540

[46] Majewski T, Yao H, Bondaruk J, Chung W, Lee S, Lee JG, et al. Whole-Organ Genomic Characterization of Mucosal Field Effects Initiating Bladder Carcinogenesis. Cell Rep. 2019;26(8):2241–56 e4. Epub 2019/02/21. doi: 10.1016/j.celrep.2019.01.095 . 30784602

[47] Pellini B, Pejovic N, Wenjia F, Earland N, Harris P, Usmani A, et al. ctDNA MRD detection and personalized oncogenomic analysis in oligometastatic colorectal cancer from plasma and urine. JCO Precis Oncol. 2021;5:378–89. doi: 10.1200/PO.20.00276 34250420 PMC8232837

[48] Pietzak EJ, Bagrodia A, Cha EK, Drill EN, Iyer G, Isharwal S, et al. Next-generation Sequencing of Nonmuscle Invasive Bladder Cancer Reveals Potential Biomarkers and Rational Therapeutic Targets. Eur Urol. 2017;72(6):952–9. Epub 2017/06/07. doi: 10.1016/j.eururo.2017.05.032 ; PubMed Central PMCID: PMC6007852. 28583311 PMC6007852

[49] Squibb Bristol-Myers. An Investigational Immuno-therapy Study of Nivolumab, Compared to Placebo, in Patients With Bladder or Upper Urinary Tract Cancer, Following Surgery to Remove the Cancer (CheckMate 274). [ClinicalTrials.gov identifier NCT02632409]. National Institutes of Health, ClinicalTrials.gov. [cited 2020]. Available from: https://clinicaltrials.gov/ct2/show/NCT02632409. Accessed 2021 Jan 11.

[50] National Cancer Institute (NCI) and Alliance for Clinical Trials in Oncology. Testing MK-3475 (Pembrolizumab) After Surgery for Localized Muscle-Invasive Bladder Cancer and Locally Advanced Urothelial Cancer (AMBASSADOR). [ClinicalTrials.gov identifier NCT03244483]. National Institutes of Health, ClinicalTrials.gov. Available from: https://clinicaltrials.gov/ct2/show/NCT03244384. Accessed 2021 Jan 11.

[51] Galsky MD, Arija JÁ A, Bamias A, Davis ID, De Santis M, Kikuchi E, et al. Atezolizumab with or without chemotherapy in metastatic urothelial cancer (IMvigor130): a multicentre, randomised, placebo-controlled phase 3 trial. Lancet. 2020;395(10236):1547–57. Epub 2020/05/18. doi: 10.1016/S0140-6736(20)30230-0 . 32416780

[52] Gandara DR, Paul SM, Kowanetz M, Schleifman E, Zou W, Li Y, et al. Blood-based tumor mutational burden as a predictor of clinical benefit in non-small-cell lung cancer patients treated with atezolizumab. Nat Med. 2018;24(9):1441–8. Epub 2018/08/08. doi: 10.1038/s41591-018-0134-3 . 30082870

[53] Chan TA, Yarchoan M, Jaffee E, Swanton C, Quezada SA, Stenzinger A, et al. Development of tumor mutation burden as an immunotherapy biomarker: utility for the oncology clinic. Ann Oncol. 2019;30(1):44–56. Epub 2018/11/06. doi: 10.1093/annonc/mdy495 ; PubMed Central PMCID: PMC6336005. 30395155 PMC6336005

[54] Powles TBAZ, Davarpanah N, Hussain M, Oudard S, Gschwend JE, et al. Clinical outcomes in post-operative ctDNA-positive muscle-invasive urothelial carcinoma (MIUC) patients after atezolizumab adjuvant therapy. Ann Oncol. 2020;01:2020.

[55] Galsky MD, Saci A, Szabo PM, Han GC, Grossfeld G, Collette S, et al. Nivolumab in Patients with Advanced Platinum-resistant Urothelial Carcinoma: Efficacy, Safety, and Biomarker Analyses with Extended Follow-up from CheckMate 275. Clin Cancer Res. 2020;26(19):5120–8. Epub 2020/06/14. doi: 10.1158/1078-0432.CCR-19-4162 . 32532789 PMC8166422

[56] Tate JG, Bamford S, Jubb HC, Sondka Z, Beare DM, Bindal N, et al. COSMIC: the Catalogue Of Somatic Mutations In Cancer. Nucleic Acids Res. 2019;47(D1):D941–D7. Epub 2018/10/30. doi: 10.1093/nar/gky1015 ; PubMed Central PMCID: PMC6323903. 30371878 PMC6323903

[57] Andre F, Ciruelos E, Rubovszky G, Campone M, Loibl S, Rugo HS, et al. Alpelisib for PIK3CA-Mutated, Hormone Receptor-Positive Advanced Breast Cancer. N Engl J Med. 2019;380(20):1929–40. Epub 2019/05/16. doi: 10.1056/NEJMoa1813904 . 31091374

[58] Juric D, Janku F, Rodon J, Burris HA, Mayer IA, Schuler M, et al. Alpelisib Plus Fulvestrant in PIK3CA-Altered and PIK3CA-Wild-Type Estrogen Receptor-Positive Advanced Breast Cancer: A Phase 1b Clinical Trial. JAMA Oncol. 2019;5(2):e184475. Epub 2018/12/14. doi: 10.1001/jamaoncol.2018.4475; PubMed Central PMCID: PMC6439561. 30543347 PMC6439561

[59] Franz DN, Belousova E, Sparagana S, Bebin EM, Frost M, Kuperman R, et al. Efficacy and safety of everolimus for subependymal giant cell astrocytomas associated with tuberous sclerosis complex (EXIST-1): a multicentre, randomised, placebo-controlled phase 3 trial. Lancet. 2013;381(9861):125–32. Epub 2012/11/20. doi: 10.1016/S0140-6736(12)61134-9 . 23158522

[60] Springer SU, Chen CH, Rodriguez Pena MDC, Li L, Douville C, Wang Y, et al. Non-invasive detection of urothelial cancer through the analysis of driver gene mutations and aneuploidy. Elife. 2018;7. Epub 2018/03/21. doi: 10.7554/eLife.32143; PubMed Central PMCID: PMC5860864. 29557778 PMC5860864

[61] Chen X, Zhang J, Ruan W, Huang M, Wang C, Wang H, et al. Urine DNA methylation assay enables early detection and recurrence monitoring for bladder cancer. J Clin Invest. 2020;130(12):6278–89. Epub 2020/08/21. doi: 10.1172/JCI139597 ; PubMed Central PMCID: PMC7685755. 32817589 PMC7685755

[62] Mar N, Dayyani F. Management of Urothelial Bladder Cancer in Clinical Practice: Real-World Answers to Difficult Questions. J Oncol Pract. 2019;15(8):421–8. Epub 2019/08/14. doi: 10.1200/JOP.19.00215 . 31404517

[63] Dobruch J, Daneshmand S, Fisch M, Lotan Y, Noon AP, Resnick MJ, et al. Gender and Bladder Cancer: A Collaborative Review of Etiology, Biology, and Outcomes. Eur Urol. 2016;69(2):300–10. Epub 2015/09/09. doi: 10.1016/j.eururo.2015.08.037 . 26346676

[64] Siefker-Radtke AO, Mellado B, Decaestecker K, Burke JM, O’Hagan A, Avadhani AN, et al. A phase 2 study of JNJ-42756493, a pan-FGFR tyrosine kinase inhibitor, in patients (pts) with metastatic or unresectable urothelial cancer (UC) harboring FGFR gene alterations. J Clin Oncol. 2016;34(15_suppl):TPS4575–TPS. doi: 10.1200/JCO.2016.34.15_suppl.TPS4575

[65] Loriot Y, Necchi A, Park SH, Garcia-Donas J, Huddart R, Burgess E, et al. Erdafitinib in Locally Advanced or Metastatic Urothelial Carcinoma. N Engl J Med. 2019;381(4):338–48. Epub 2019/07/25. doi: 10.1056/NEJMoa1817323 . 31340094

[66] Panebianco V, Narumi Y, Altun E, Bochner BH, Efstathiou JA, Hafeez S, et al. Multiparametric Magnetic Resonance Imaging for Bladder Cancer: Development of VI-RADS (Vesical Imaging-Reporting And Data System). Eur Urol. 2018;74(3):294–306. Epub 2018/05/15. doi: 10.1016/j.eururo.2018.04.029 ; PubMed Central PMCID: PMC6690492. 29755006 PMC6690492

[67] Bailey MH, Tokheim C, Porta-Pardo E, Sengupta S, Bertrand D, Weerasinghe A, et al. Comprehensive Characterization of Cancer Driver Genes and Mutations. Cell. 2018;173(2):371–85 e18. Epub 2018/04/07. doi: 10.1016/j.cell.2018.02.060 ; PubMed Central PMCID: PMC6029450. 29625053 PMC6029450

[68] Alexandrov LB, Nik-Zainal S, Wedge DC, Aparicio SA, Behjati S, Biankin AV, et al. Signatures of mutational processes in human cancer. Nature. 2013;500(7463):415–21. Epub 2013/08/16. doi: 10.1038/nature12477 ; PubMed Central PMCID: PMC3776390. 23945592 PMC3776390

