# Correction: Urine tumor DNA detection of minimal residual disease in muscle-invasive bladder cancer treated with curative-intent radical cystectomy: A cohort study

**DOI:** 10.1371/journal.pmed.1003876

**Published:** 2021-12-14

**Authors:** Pradeep S. Chauhan, Kevin Chen, Ramandeep K. Babbra, Wenjia Feng, Nadja Pejovic, Armaan Nallicheri, Peter K. Harris, Katherine Dienstbach, Andrew Atkocius, Lenon Maguire, Faridi Qaium, Jeffrey J. Szymanski, Brian C. Baumann, Li Ding, Dengfeng Cao, Melissa A. Reimers, Eric H. Kim, Zachary L. Smith, Vivek K. Arora, Aadel A. Chaudhuri

In the abstract, the number of patients in the pCR and no pCR groups are interchanged. It should be *n* = 26 for patients classified as having residual disease detected in their surgical sample, and *n* = 16 for those who achieved a pCR. Additionally, the DRR acronym in the abstract should not be present. Please see below for the corrected abstract:

## Background

The standard of care treatment for muscle-invasive bladder cancer (MIBC) is radical cystectomy, which is typically preceded by neoadjuvant chemotherapy. However, the inability to assess minimal residual disease (MRD) noninvasively limits our ability to offer bladder-sparing treatment. Here, we sought to develop a liquid biopsy solution via urine tumor DNA (utDNA) analysis.

## Methods and findings

We applied urine Cancer Personalized Profiling by Deep Sequencing (uCAPP-Seq), a targeted next-generation sequencing (NGS) method for detecting utDNA, to urine cell-free DNA (cfDNA) samples acquired between April 2019 and November 2020 on the day of curative-intent radical cystectomy from 42 patients with localized bladder cancer. The average age of patients was 69 years (range: 50 to 86), of whom 76% (32/42) were male, 64% (27/42) were smokers, and 76% (32/42) had a confirmed diagnosis of MIBC. Among MIBC patients, 59% (19/32) received neoadjuvant chemotherapy. utDNA variant calling was performed noninvasively without prior sequencing of tumor tissue. The overall utDNA level for each patient was represented by the non-silent mutation with the highest variant allele fraction after removing germline variants. Urine was similarly analyzed from 15 healthy adults. utDNA analysis revealed a median utDNA level of 0% in healthy adults and 2.4% in bladder cancer patients. When patients were classified as those who had residual disease detected in their surgical sample (*n* = 26) compared to those who achieved a pathologic complete response (pCR; *n* = 16), median utDNA levels were 4.3% vs. 0%, respectively (*p* = 0.002). Using an optimal utDNA threshold to define MRD detection, positive utDNA MRD detection was highly correlated with the absence of pCR (*p* < 0.001) with a sensitivity of 81% and specificity of 81%. Leave-one-out cross-validation applied to the prediction of pathologic response based on utDNA MRD detection in our cohort yielded a highly significant accuracy of 81% (*p* = 0.007). Moreover, utDNA MRD-positive patients exhibited significantly worse progression-free survival (PFS; HR = 7.4; 95% CI: 1.4–38.9; *p* = 0.02) compared to utDNA MRD-negative patients. Concordance between urine- and tumor-derived mutations, determined in 5 MIBC patients, was 85%. Tumor mutational burden (TMB) in utDNA MRD-positive patients was inferred from the number of non-silent mutations detected in urine cfDNA by applying a linear relationship derived from The Cancer Genome Atlas (TCGA) whole exome sequencing of 409 MIBC tumors. We suggest that about 58% of these patients with high inferred TMB might have been candidates for treatment with early immune checkpoint blockade. Study limitations included an analysis restricted only to single-nucleotide variants, survival differences diminished by surgery, and a low number of DNA damage response mutations detected after neoadjuvant chemotherapy at the MRD time point.

## Conclusions

utDNA MRD detection prior to curative-intent radical cystectomy for bladder cancer correlated significantly with pathologic response, which may help select patients for bladder-sparing treatment. utDNA MRD detection also correlated significantly with PFS. Furthermore, utDNA can be used to noninvasively infer TMB, which could facilitate personalized immunotherapy for bladder cancer in the future.
