# Supplementary figures and images for: Urine tumor DNA detection of minimal residual disease in muscle-invasive bladder cancer treated with curative-intent radical cystectomy: A cohort study
Source: PLoS Med. 2021 Aug 31;18(8):e1003732. doi: 10.1371/journal.pmed.1003732 (PMC8407541; doi:10.1371/journal.pmed.1003732)

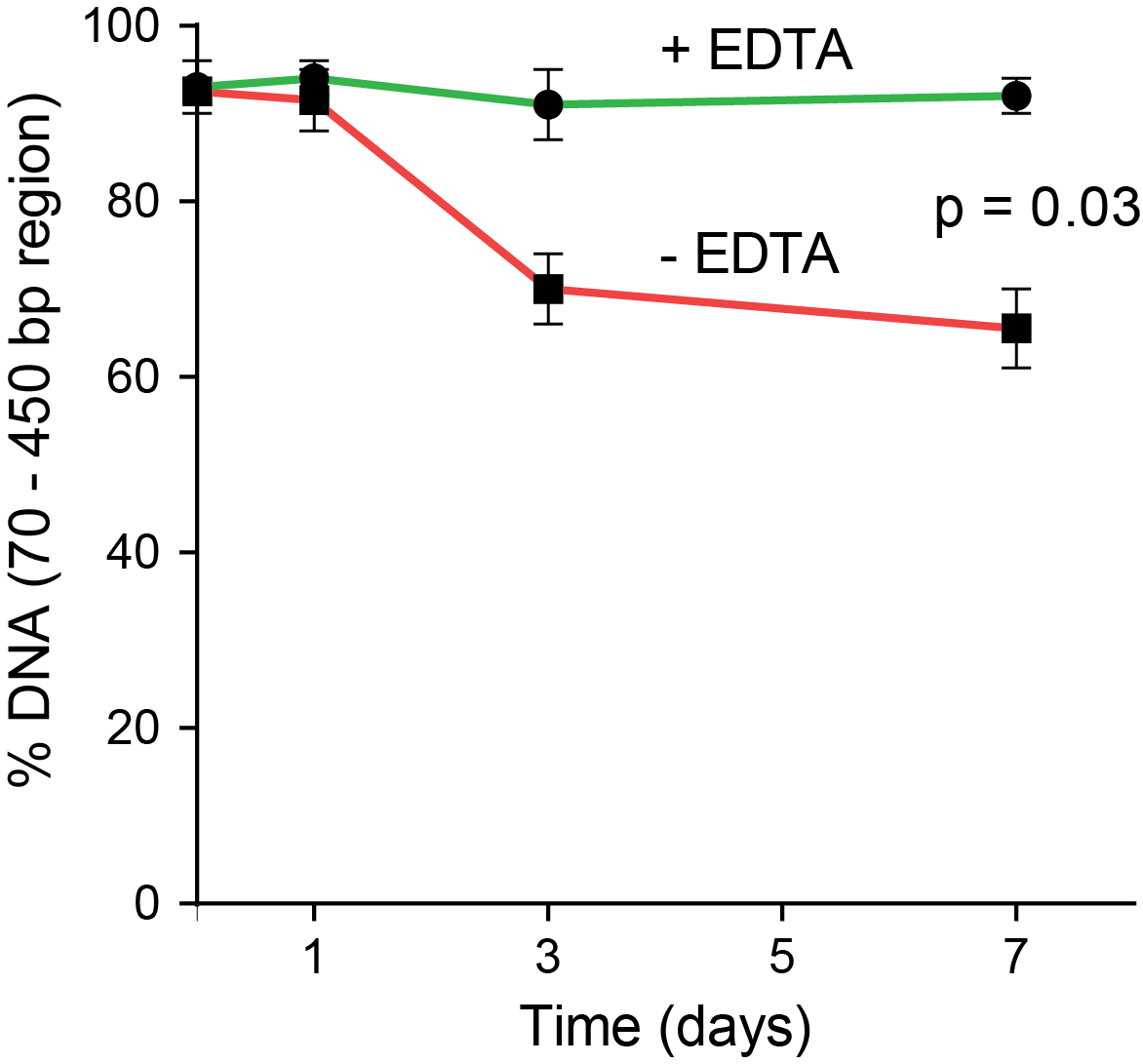

Supplement: S1 Fig — Technical experiment to evaluate the stability of urine cfDNA with and without the addition of EDTA. Pooled urine from a healthy volunteer was stored for 7 days with versus without 10 mM EDTA at room temperature. Urine cfDNA was extracted at days 0, 1, 3, and 7 after commencing storage. The effect of EDTA on urine cfDNA degradation was evaluated based on the percentage of DNA in the 70–450 bp size range on Bioanalyzer eletropherogram. The data presented here are mean ± SEM of 2 replicates per time point. The p-value was calculated by unpaired Student t test at the last time point. bp, base pair; cfDNA, cell-free DNA; SEM, standard error of the mean. (TIF) [file pmed.1003732.s002.tif]

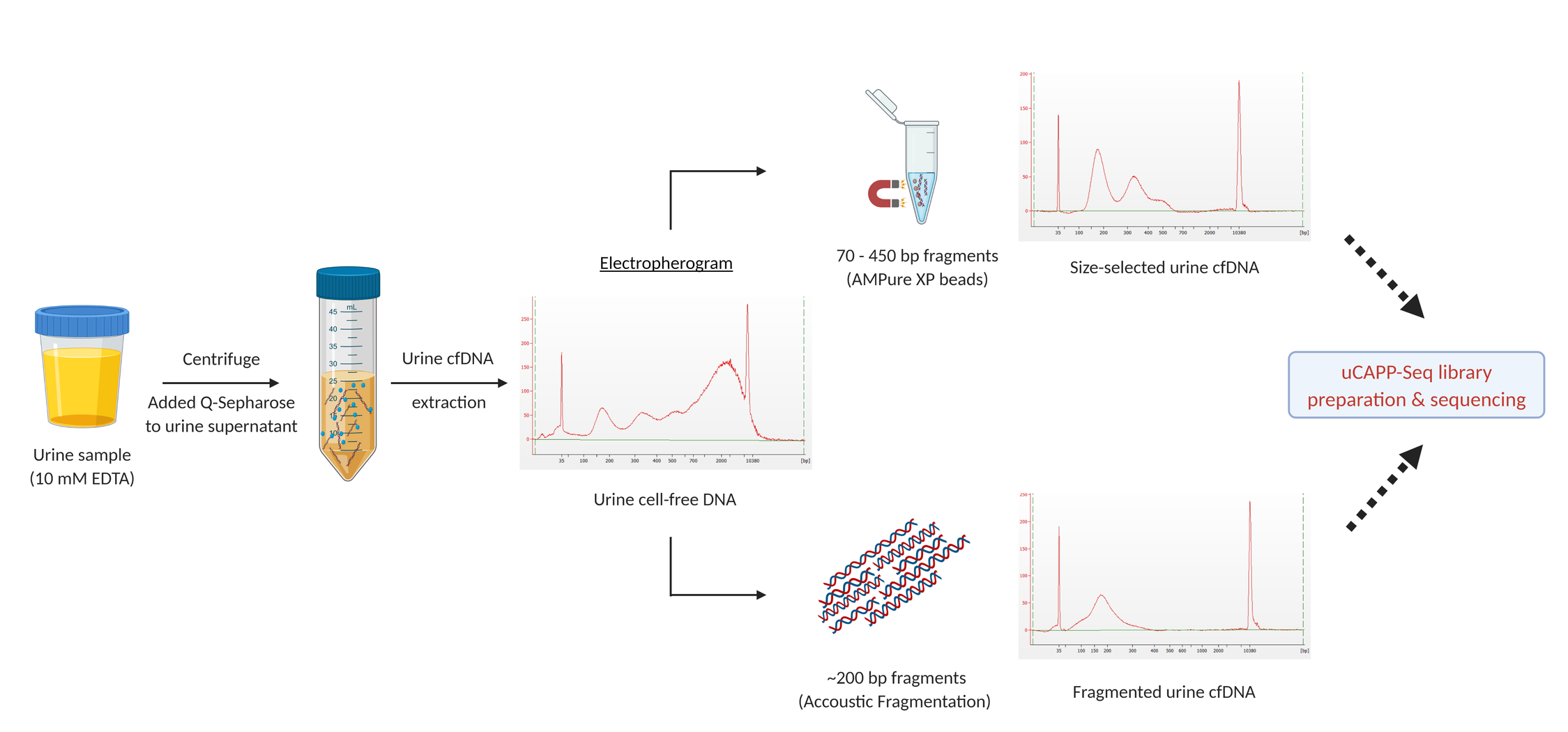

Supplement: S2 Fig — Representative electropherograms are also shown, all from the same MIBC patient’s urine sample. bp, base pair; cfDNA, cell-free DNA; MIBC, muscle-invasive bladder cancer; mM, millimolar; uCAPP-Seq, urine Cancer Personalized Profiling by Deep Sequencing. (TIF) [file pmed.1003732.s003.tif]

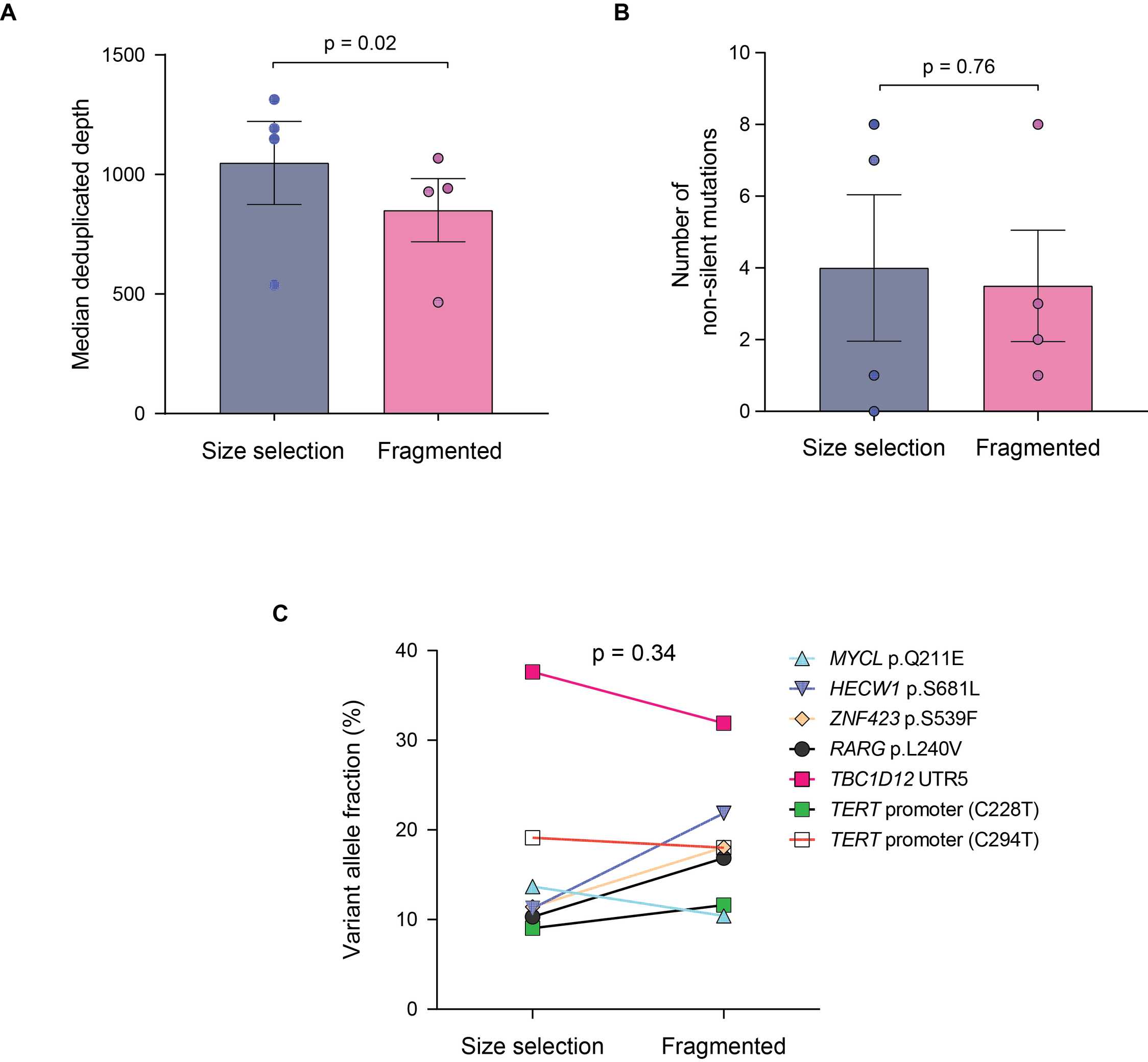

Supplement: S3 Fig — (A) Median deduplicated sequencing depths, (B) Number of non-silent mutations, (C) Mutant allele fraction levels in 4 MIBC urine cfDNA samples that underwent in vitro size selection or acoustic DNA fragmentation (see S2 Fig for schema). Data values are depicted as points, column heights represent averages, and error bars represent standard error of the mean. VAF comparisons in (C) are also shown with connecting lines. The p-values were calculated using the paired Student t test method. bp, base pair; cfDNA, cell-free DNA; MIBC, muscle-invasive bladder cancer; VAF, variant allele fraction. (TIF) [file pmed.1003732.s004.tif]

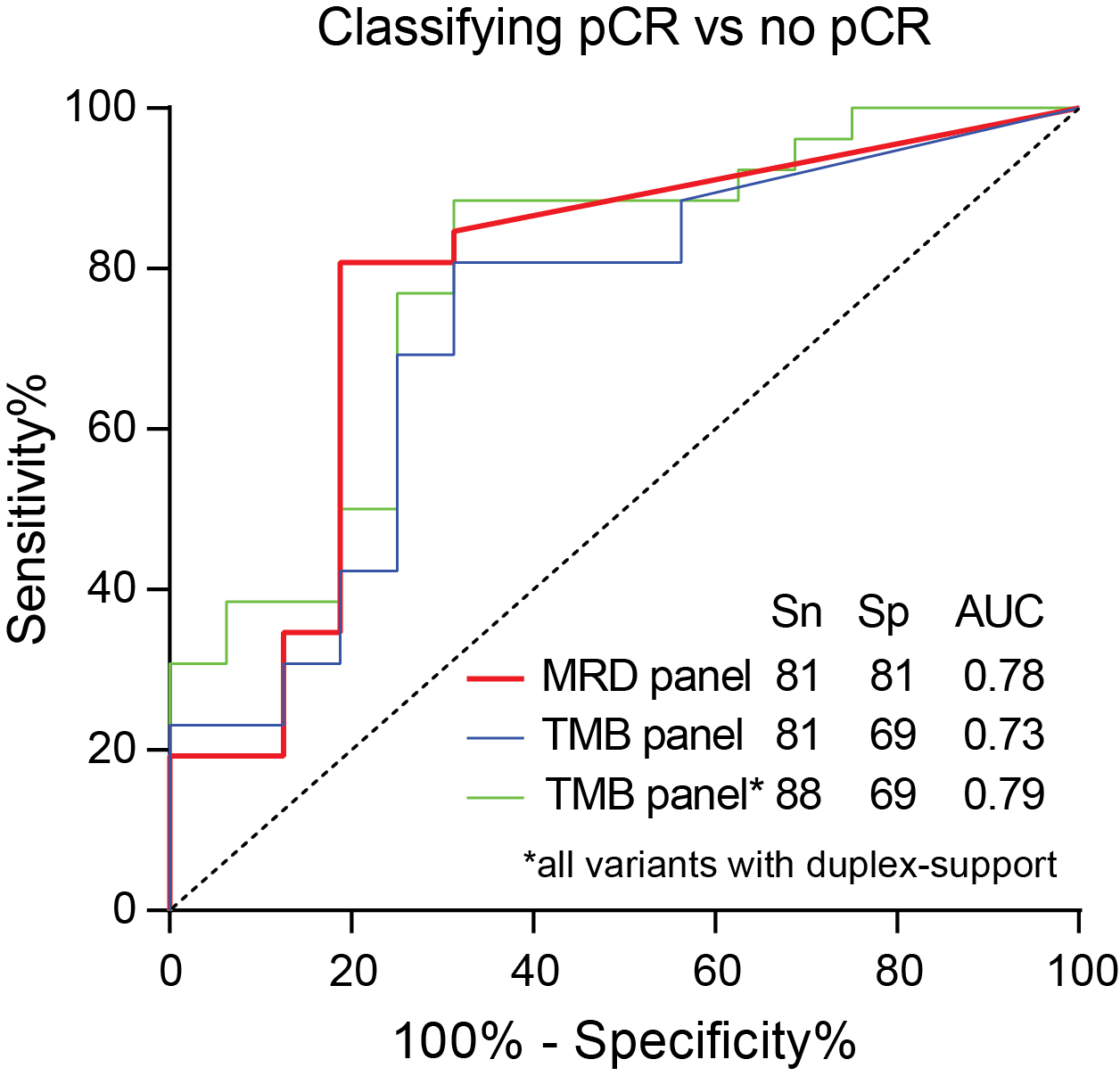

Supplement: S4 Fig — The red curve shows variant calling of non-silent, duplex-supported mutations in the MRD panel. The blue curve shows variant calling of non-silent, duplex-supported mutations in the TMB panel. The green curve shows variant calling of all mutations (silent or non-silent) with duplex support in the TMB panel. The AUC, as well as the optimal sensitivity and specificity values corresponding to Youden J statistic, is shown. AUC, area under the curve; MRD, minimal residual disease; pCR, pathologic complete response; ROC, receiver operating characteristic; Sn, sensitivity; Sp, specificity; TMB, tumor mutational burden. (TIF) [file pmed.1003732.s005.tif]

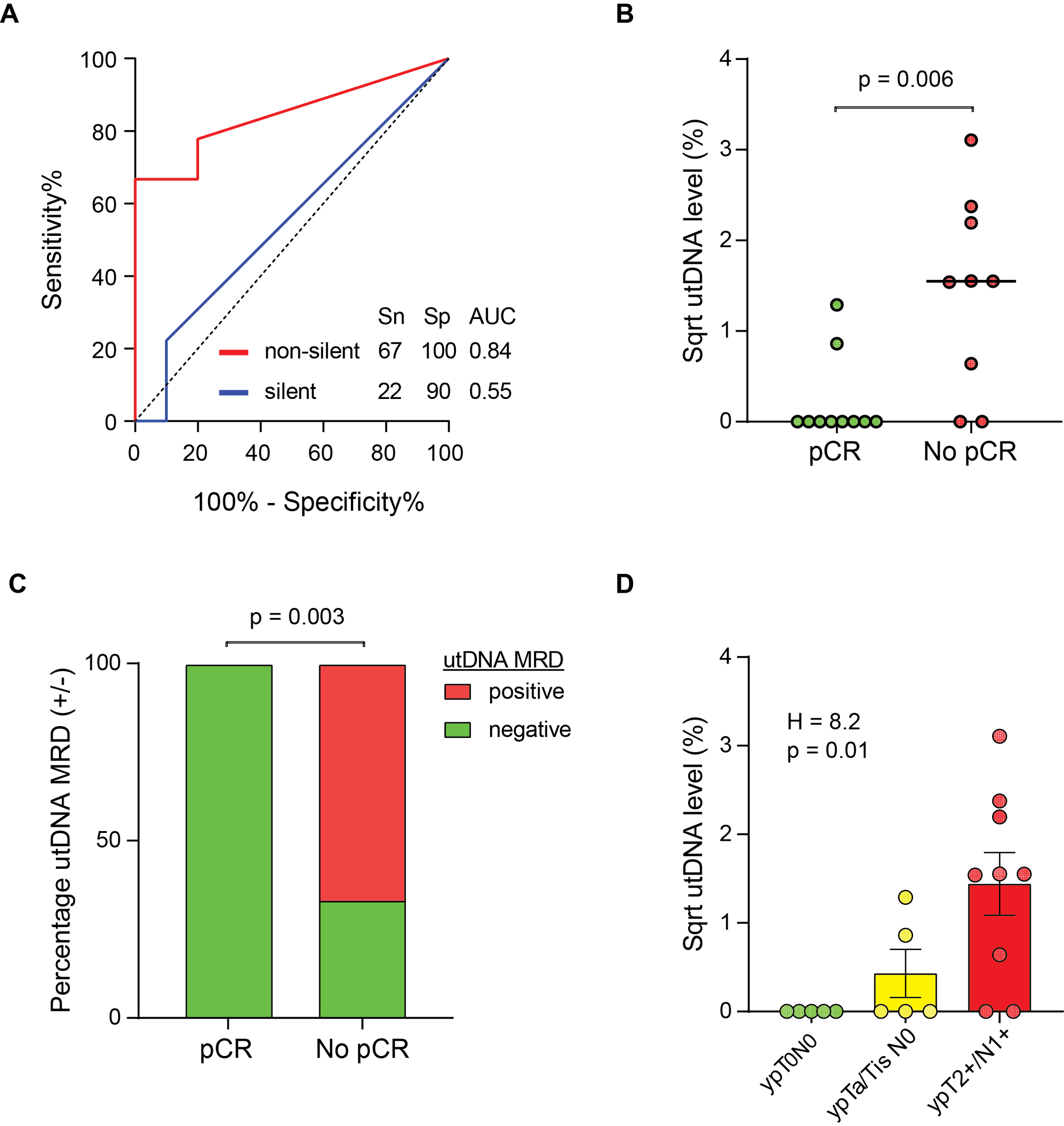

Supplement: S5 Fig — (A) ROC analysis classifying MIBC patients (either pCR or no pCR) who received neoadjuvant chemotherapy before radical cystectomy based on non-silent versus silent mutations detected in preoperative urine cfDNA. The AUC, as well as the optimal sensitivity and specificity values corresponding to Youden J statistic, is shown. (B) Scatter plot of utDNA levels (highest non-silent VAF per patient at the preoperative time point) after square root transformation in patients who achieved pCR versus those with no pCR following neoadjuvant chemotherapy. The p-value was calculated using Mann–Whitney U test. (C) Stacked bar plots showing proportions of each group with positive or negative utDNA MRD detection after neoadjuvant chemotherapy, determined by the optimal utDNA threshold (VAF: 2.3%) that classified patients with no pCR from healthy adults in the full patient cohort as described in the Methods. The p-value was calculated using Fisher exact test. (D) Scatter plot of utDNA levels after square root transformation in patients who received neoadjuvant chemotherapy, grouped by pathologic stage. Column heights depict mean values, and error bars represent the standard error of the mean. The Kruskal–Wallis H statistic and p-value are shown in the upper left. There were no patients with ypT1 disease. AUC, area under the curve; cfDNA, cell-free DNA; MIBC, muscle-invasive bladder cancer; MRD, minimal residual disease; pCR, pathologic complete response; ROC, receiver operating characteristic; Sn, sensitivity; Sp, specificity; Sqrt, square root; utDNA, urine tumor DNA; VAF, variant allele fraction. (TIF) [file pmed.1003732.s006.tif]

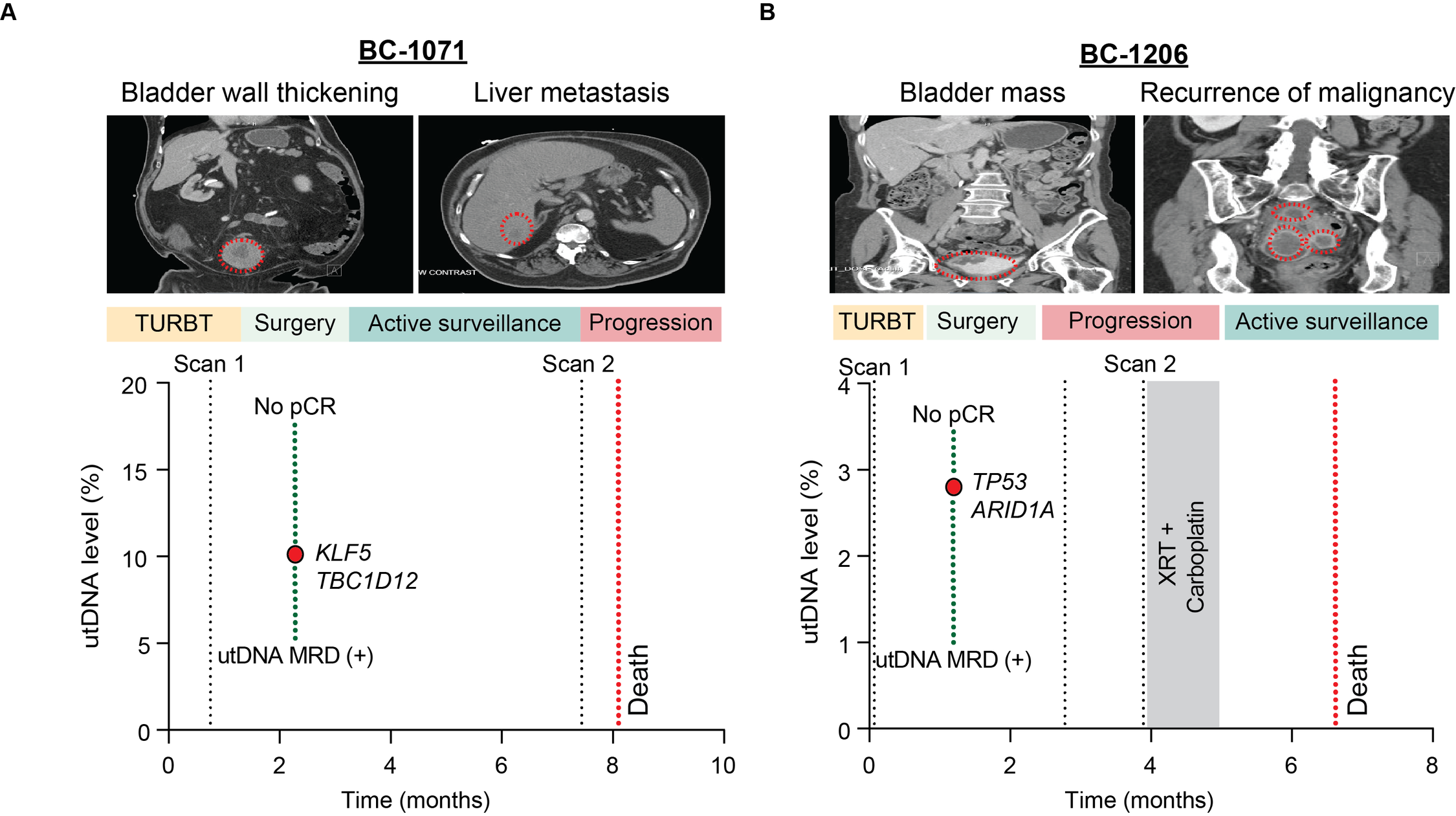

Supplement: S6 Fig — (A) Clinical vignette of MIBC patient BC-1071 who was utDNA MRD (+) at the preoperative time point with a utDNA level of 10.1%, which was concordant with no pCR on surgical pathology. The patient progressed 160 days later and died shortly thereafter. (B) Clinical vignette of MIBC patient BC-1206 who was utDNA MRD (+) at the preoperative time point with a utDNA level of 2.8%, which was concordant with no pCR on surgical pathology. The patient progressed at 46 days after radical cystectomy, received chemoradiation to treat the recurrence, but, nonetheless, died at 164 days. MIBC, muscle-invasive bladder cancer; MRD, minimal residual disease; pCR, pathologic complete response; TURBT, transurethral resection of bladder tumor; utDNA, urine tumor DNA. (TIF) [file pmed.1003732.s007.tif]

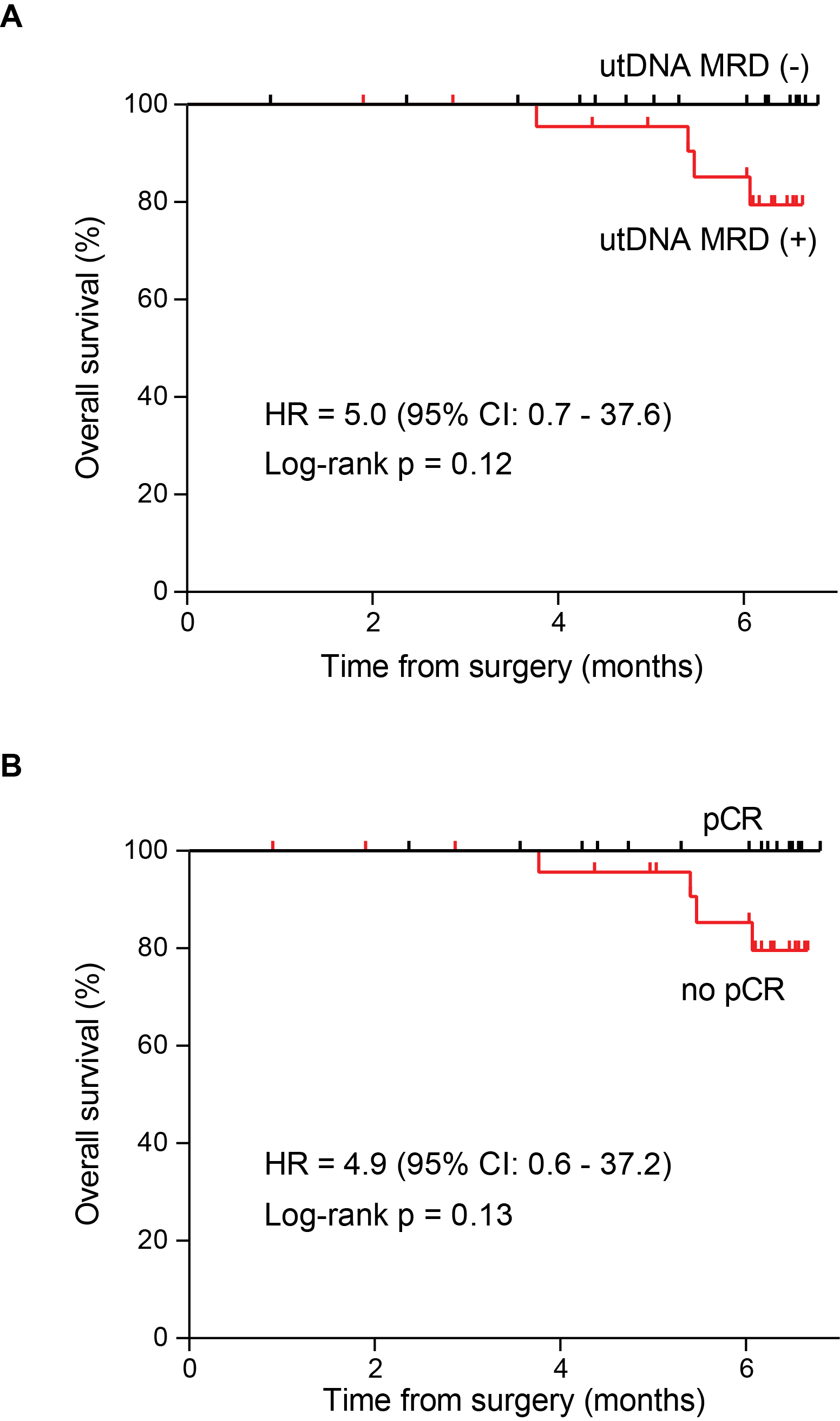

Supplement: S7 Fig — OS analysis according to utDNA MRD detection at the preoperative time point (A) and pathologic response assessed in the radical cystectomy specimen (B). HRs and p-values were calculated using the Mantel–Haenszel and Mantel–Cox methods, respectively. CI, confidence interval; HR, hazard ratio; MRD, minimal residual disease; OS, overall survival; pCR, pathologic complete response, utDNA, urine tumor DNA. (TIF) [file pmed.1003732.s008.tif]
